# Short- and long-term effects of radiation exposure at low dose and low dose rate in normal human VH10 fibroblasts

**DOI:** 10.3389/fpubh.2023.1297942

**Published:** 2023-12-15

**Authors:** Pamela Akuwudike, Milagrosa López-Riego, Michal Marczyk, Zuzana Kocibalova, Fabian Brückner, Joanna Polańska, Andrzej Wojcik, Lovisa Lundholm

**Affiliations:** ^1^Centre for Radiation Protection Research, Department of Molecular Biosciences, The Wenner-Gren Institute, Stockholm University, Stockholm, Sweden; ^2^Department of Data Science and Engineering, Silesian University of Technology, Gliwice, Poland; ^3^Yale Cancer Center, Yale School of Medicine, New Haven, CT, United States; ^4^Institute of Biology, Jan Kochanowski University, Kielce, Poland

**Keywords:** low dose, low dose rate, dose and dose rate effectiveness factor, radiation carcinogenesis, fibroblasts

## Abstract

**Introduction:**

Experimental studies complement epidemiological data on the biological effects of low doses and dose rates of ionizing radiation and help in determining the dose and dose rate effectiveness factor.

**Methods:**

Human VH10 skin fibroblasts exposed to 25, 50, and 100 mGy of ^137^Cs gamma radiation at 1.6, 8, 12 mGy/h, and at a high dose rate of 23.4 Gy/h, were analyzed for radiation-induced short- and long-term effects. Two sample cohorts, i.e., discovery (*n* = 30) and validation (*n* = 12), were subjected to RNA sequencing. The pool of the results from those six experiments with shared conditions (1.6 mGy/h; 24 h), together with an earlier time point (0 h), constituted a third cohort (*n* = 12).

**Results:**

The 100 mGy-exposed cells at all abovementioned dose rates, harvested at 0/24 h and 21 days after exposure, showed no strong gene expression changes. *DMXL2*, involved in the regulation of the NOTCH signaling pathway, presented a consistent upregulation among both the discovery and validation cohorts, and was validated by qPCR. Gene set enrichment analysis revealed that the NOTCH pathway was upregulated in the pooled cohort (*p* = 0.76, normalized enrichment score (NES) = 0.86). Apart from upregulated apical junction and downregulated DNA repair, few pathways were consistently changed across exposed cohorts. Concurringly, cell viability assays, performed 1, 3, and 6 days post irradiation, and colony forming assay, seeded just after exposure, did not reveal any statistically significant early effects on cell growth or survival patterns. Tendencies of increased viability (day 6) and reduced colony size (day 21) were observed at 12 mGy/h and 23.4 Gy/min. Furthermore, no long-term changes were observed in cell growth curves generated up to 70 days after exposure.

**Discussion:**

In conclusion, low doses of gamma radiation given at low dose rates had no strong cytotoxic effects on radioresistant VH10 cells.

## Introduction

Environmental and occupational exposures mostly involve low doses of ionizing radiation (IR) delivered at low dose rates, a scenario for which the risk of stochastic effects is poorly defined given the larger uncertainties from epidemiological data (1, 2). Low dose rates categorize exposures with <0.1 mGy/min averaged over 1 h (<6 mGy/h), and low doses are defined as <100 mGy (3). Radiation protection standards are largely based on the Life Span Study (LSS) cohort of atomic bomb survivors of Hiroshima and Nagasaki, which despite being the major epidemiological source of information on radiation-induced effects (4), comprises individuals exposed at a high dose rate (5). Besides, LSS-derived risk estimates are largely impacted by those who received moderately to high doses (6). The assumption of a decrease in cancer induction by half when low linear energy transfer radiation is delivered at a low total dose and dose rate, as recommended by the International Commission on Radiological Protection (ICRP) through the use of a dose and dose rate effectiveness factor (DDREF) of 2 (7), is not or only partially supported by other organizations (3, 8). Experimental studies complement epidemiological data on the dose and dose rate effect, providing dose responses among molecular biomarkers and mechanistic insight which can be used for adverse outcome pathway development and risk assessment (9–13).

To ultimately understand the potential difference in biological effectiveness of low doses delivered at low dose rates, it is important to simultaneously investigate the short- and long-term alterations using different endpoints. The best radiation protection assurance will likely not involve a definitive unique biomarker but a combination of novel and already identified signatures (14–16). To date, no biomarker fulfils the criteria to be regarded as an ideal biomarker for assessing exposure, effect or susceptibility to low dose radiation exposure, as concluded by the European Low Dose Research towards Multidisciplinary Integration (DoReMi) project (14). Notably, most IR biomarkers have been characterized at early time points after high doses of low-LET radiation due to practical reasons (17), as it is the most extended radiation source setting in external beam radiotherapy (18). So specific and sensitive biomarkers of exposure to low doses and dose rates are still needed. Global gene expression changes provide insight into biological responses to IR (19–22) and may serve as sensitive biomarkers of exposure. IR-induced genes are often involved in cell cycle regulation, apoptosis, proliferation, DNA damage response and repair, DNA methylation and chromatin remodeling (23–26).

Normal human fibroblasts are well known for their value as a model system of normal cell radiosensitivity to analyze the early and long-term effects of ionizing radiation (27). For this reason, and because skin, the largest organ in the body, is inevitably exposed during external IR exposures, several studies have focused on the radiation-induced transcriptomic changes that occur in cultured primary human fibroblasts, skin models or patient skin biopsies (10, 28–46), for a review see (19). Gene expression profiles are dynamic and quantitatively and qualitatively different at low doses as compared to high doses, with the time point of maximum number of differentially expressed genes (DEGs) after low doses varying according to different studies (28–33). At low doses, human fibroblasts show enrichment for cell–cell signaling and DNA damage functional groups, while at high doses, apoptosis and cell proliferation genes dominate the response (28). Using a skin model, Ray et al. showed that a low dose of 100 mGy primarily triggers adaptive responses, while 1 Gy activated cell cycle processes (34). Besides, 100 mGy delivered at 63 mGy/min (3,780 mGy/h) triggers changes in apoptosis, cell proliferation, epithelial-to-mesenchymal transition (EMT), stress response and nitric oxide signaling pathways in dermal fibroblasts from a human 3D-skin model (10). Both dose and dose rate are crucial determinants of the biological response to IR (47). IMR-90 lung fibroblasts showed distinct gene expression profiles in one third of the DEGs 2 h after exposure to either 1.0 Gy/min or 0.7 mGy/min (60,000 mGy/h or 42 mGy/h), at the same dose level of 1 Gy (44). However, unlike other cell types, such as human leukemia (48) or human glioblastoma (49) cell lines, for which the dose rate effect on gene expression changes has been investigated, primary human fibroblasts or skin models have not received as much attention in this regard, or the dose rate effect has been studied in the high dose range (43, 44), with few exceptions (50).

Here, we studied the gene expression and cytotoxic effects resulting from radiation exposure at low doses and low dose rates in VH10 fibroblasts. These cells are not transformed and represent a valid model to study IR effects on normal cells. Global gene expression was analyzed by RNA-seq in two independent experimental cohorts: an initial discovery and a second validation cohort. We screened for early and late gene expression markers of exposure to 100 mGy at 1.6, 8, and 12 mGy/h as well as 23.4 Gy/h by RNA-seq. Short- and long-term effects of low dose and low dose rate (LDLDR) exposure were additionally analyzed at 25, 50, and 100 mGy at all abovementioned dose rates using two types of cell viability assays and the colony forming assay. Our results suggest weak or undetectable effects of low doses of gamma radiation delivered at low dose rates in VH10 fibroblasts.

## Materials and methods

### Cell culture

Normal human foreskin fibroblasts (VH10) donated by Leiden University were cultured in Dulbecco’s modified minimum essential medium (DMEM) (Sigma-Aldrich, Germany), supplemented with 10% bovine calf serum (HyClone, Thermo Fisher Scientific, Waltham, MA, United States) and 1% penicillin–streptomycin (10.000 U penicillin and 10 mg streptomycin/mL, Sigma-Aldrich) at 37°C and 5% CO_2_. All experiments were started with fibroblasts at passage 7 (P7), grown to 80% confluence before the start of each experiment. VH10 fibroblasts were passaged weekly in 175 cm^2^ flasks at a seeding density of 5.0 × 10^5^ cells.

### Low dose and low dose rate radiation exposure

**Figure 1:**
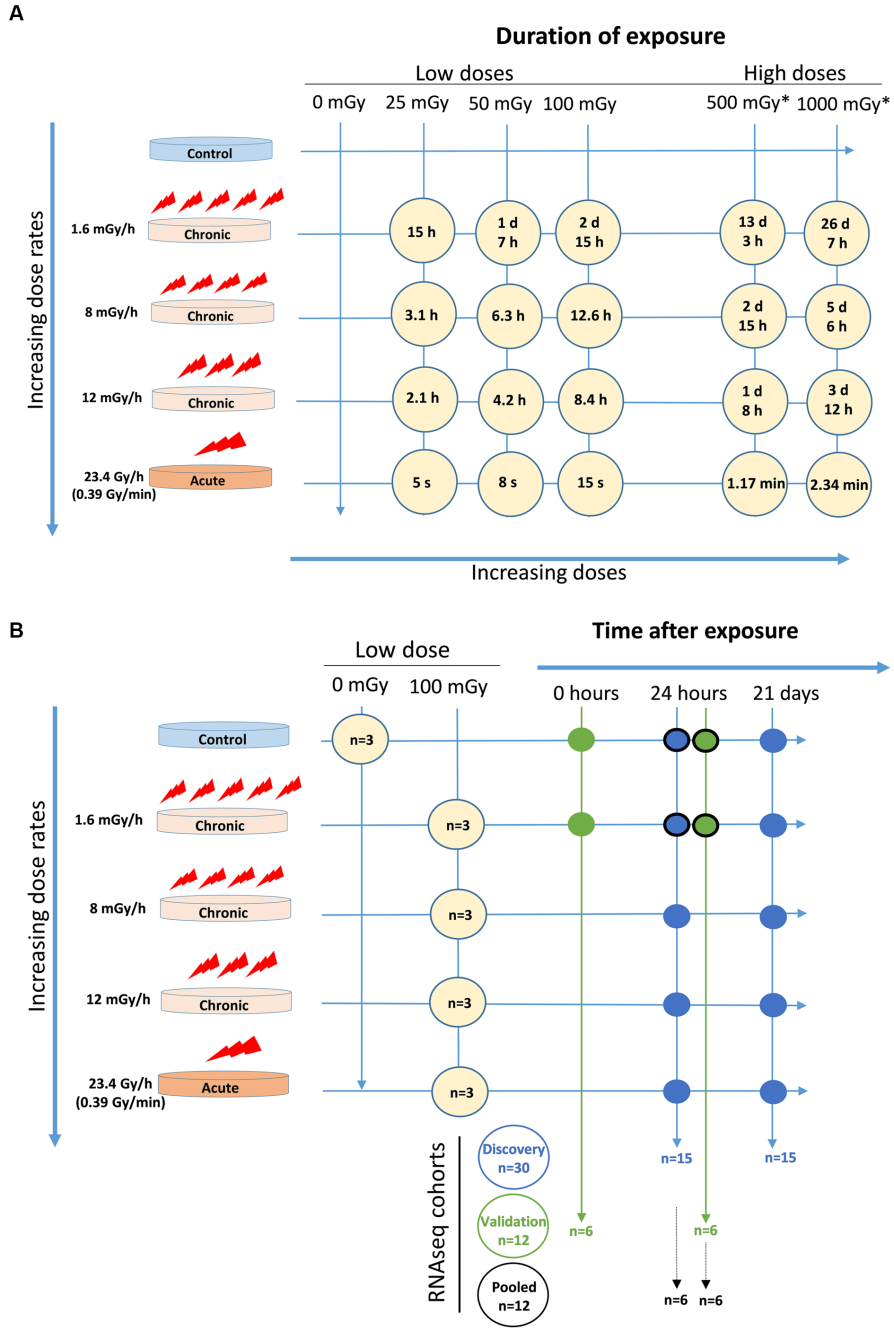
Experimental setup. **(A)** Exposure time for each dose at each dose rate. *These doses were only indicated in the agarose overlay colony forming assay. **(B)** Cell cohorts for RNAseq with number of samples (*n*) and conditions included in discovery cohort (blue circles), validation cohort (green circles), and pooled cohort (blue and green circles with black edge).

Cells were exposed to low doses, 25, 50, and 100 mGy, of gamma radiation from a ^137^Cs source at the low dose rates of 1.6, 8, and 12 mGy/h using a custom-made radiation facility available at Stockholm University, Sweden (51). The facility is composed of a 370 GBq source (as of June 2007) positioned below an incubator and covered with a 5 mm lead plate. The different dose rates were achieved by modifying the distance to the source inside the incubator and additional lead filtering. RNA-seq was performed to assess global early and late gene expression changes in VH10 fibroblasts induced by exposure to 100 mGy of ^137^Cs gamma radiation at 1.6, 8, and 12 mGy/h as well as 23.4 Gy/h (Figures 1A,B). VH10 cells were also exposed to 25, 50, 100, 500, and 1,000 mGy, at 1.6, 8, and 12 mGy/h and at a high dose rate of 23.4 Gy/h (0.39 Gy/min), using Scanditronix (Uppsala, Sweden), for colony formation and cell viability (25, 50, and 100 mGy doses only) assays (Figure 1A). Irradiation was carried out at 37°C for chronic exposure and at room temperature at the acute dose rate. Non-irradiated controls were treated in a similar manner as irradiated samples (Figure 1A).

### RNA sequencing experiment and cell cohorts

Two cohorts consisting of three independent experiments for each experimental condition, and a pool of these six experiments, were analysed (Figure 1B). The first (discovery) cohort included data from 30 samples, where two factors were considered, namely radiation dose rate (1.6, 8, 12, and 23.4 Gy/h) and time after exposure (24 h and 21 days), all (except controls) at a dose of 100 mGy. The second (validation) cohort comprised data from 12 samples, where the radiation dose rate was kept constant at 1.6 mGy/h while a time after exposure factor was included (0 and 24 h). For the pooled cohort analysis, sequencing data from the discovery and validation cohort (control and 1.6 mGy/h irradiated samples measured at 24 h) were considered. Consequently, the pooled cohort included 12 samples, in which two factors were analysed, namely radiation exposure and cohort, i.e., discovery or validation. RNA was extracted using the E.Z.N.A. Total RNA Kit I (Omega Biotek, United States) and RNA integrity was assessed with the help of a Bioanalyzer. RNA Integrity Number (RIN) values ≥9 indicated good quality RNA. Illumina TruSeq Stranded mRNA (using poly-A selection) libraries were obtained from total RNA samples. For the discovery cohort samples, sequencing was performed using multiplex in 0.5 lane on Illumina NovaSeq S4, PE 2 × 150bp. For the validation cohort, samples were sequenced on 0.25 lane Illumina NovaSeq 6,000 S4-300, 2 × 150bp including XP kit.

The quality of raw sequencing reads was assessed using FastqQC v0.11.5 (52). Adapter sequences were identified and removed using Trimmomatic v0.39 (53). The remaining reads were aligned to a human genome standard (hg38) using STAR v2.7.6a with two-pass mode and default parameters (54). Gene expression was quantified using RSEM v1.3.0 (55) resulting in transcripts per million (TPM) scaled up to library size. ENSEMBL release 101 was used to annotate reads within human genes. Only protein-coding genes were selected for statistical analyses, and genes not expressed in all samples within the cohort were removed. Variance stabilizing transformation was used to normalize data. A filtering threshold on mean expression across all samples was defined using GaMRed software (56) to filter away low-expressed genes and increase the power to find dose rate-dependent genes in the discovery cohort.

### RT-qPCR validation of *DMXL2*

The validation of differential *DMXL2* gene expression 24 h after exposure was performed in both discovery and validation cohorts in the control and 100 mGy-exposed samples at the lowest dose rate 1.6 mGy/h. cDNA was synthesized from the same RNA samples as used for RNA sequencing using the High-Capacity cDNA Reverse Transcription Kit (Thermo Fisher Scientific, United States) according to manufacturer’s instructions. Quantitative PCR reaction was performed in triplicates with 5× HOT FIREPol EvaGreen qPCR SuperMix (Solis Biodyne, Estonia) on a LightCycler^®^ 480 (Roche, Switzerland). Cycles were as followed: 95°C for 2 min, 45 cycles of 95°C for 15 s, 60°C for 20 s, and 72°C for 20 s. Specificity of the primers was confirmed by melting curve analysis. Relative gene expression was calculated using the 2^−ΔCt^ method with *18S* as a reference gene. Primer sequences used: *18S* forward 5′-GCTTAATTTGACTCAACACGGGA-3′; *18S* reverse 5′-AGCTATCAATCTGTCAATCCTGTCC-3′; *DMXL2* forward 5′-GCAAGTATATGCCCAGGGGTTCT; *DMXL2* reverse 5′-GCTGGAAGTGGTAGTCTCACAA-3′.

### Trypan blue exclusion assay

Cell concentration was determined using the trypan blue exclusion assay as described earlier (57) and obtained using an automated cell counter (Countess, Invitrogen, United Kingdom). Cell growth was monitored by determining the population doubling (PD) according to the following equation: PD = $\ln\left(Nt/N0\right)∕\ln$ 2, where *N*t is the total number of cells after harvesting and *N*0 is the cell number seeded. Cell growth curves were created by plotting the cumulative increase in population doubling after each passage against time in culture. The total population doubling was the sum of all population doublings.

### Agarose overlay colony formation assay

Colony formation was carried out as described previously (58). 24 h prior to radiation exposure, 500 cells were seeded in triplicates in six-well culture plates. Before radiation exposure, growth medium was discarded and replaced with 1.5 mL of 0.75% of agarose mixture consisting of 1% low melting temperature agarose (Ultrapure MB Grade, USB, United States) in distilled water and 2 × DMEM supplemented with (10% bovine calf serum and 1% penicillin–streptomycin, at a ratio 4:3). This agarose mixture was allowed to set for 7–10 min at 4°C, after which 2 mL of growth media was added over the agarose overlay. The plates were incubated at 37°C and 5% CO_2_ for 21 days. Colony fixation and staining were carried out simultaneously using a 5% Giemsa solution containing 25% methanol. The number of colonies and size of colonies were determined automatically using countPHICS program (59), and a total of five independent experiments were performed.

### MTT and resazurin reduction cell viability assay

VH10 fibroblasts were seeded at a cell density of 2,000 cells per well in a 96-well plate 24 h before radiation exposure and incubated at 37°C. MTT and resazurin reduction assay was performed at 1, 3, and 6 days after radiation. MTT assay was carried out by adding (3-4,5-dimethylthiazol-2-yl)/2, 5-diphenyl-tetrazolium bromide (MTT reagent) at a final concentration of 0.25 mg/mL, followed by a 4 h incubation. MTT reaction was stopped by adding 3% sodium dodecyl sulphate (SDS) in 0.03 M HCl. Absorbance was read at 595 nm. Resazurin reduction assay was carried out by replacing growth media with 100 μL of Dulbecco’s modified minimum essential medium (DMEM) without phenol red, containing resazurin reagent (Alamar blue) at a final concentration of 10 μg/mL. Fluorescence was measured at excitation and emission wavelength 535/590 nm (excitation/emission) at 4 h after addition of resazurin.

### Statistical analysis

In univariate analysis of RNA-seq expression data using DESeq2 R package (60), different strategies were applied: (i) each irradiated sample at a given dose rate was compared to the control, separately at each time point (this is expressed as control vs. irradiated in the figures, whereby the second term of the comparison is always compared to the first); (ii) each higher dose rate was compared to each lower dose rate, separately at each time point; (iii) the control sample at 21 days after exposure was compared to that after 24 h; (iv) two-factor analysis was performed including dose rates and time points with interactions to identify DEGs with opposite expression patterns at the two different time points. In all analyses, the significance level was set to 0.2. Additionally, gene expression changes were examined in a panel of six known radiation-responsive genes, i.e., *BBC3*, *CDKN1A*, *FDXR*, *GADD45A*, *MDM2*, and *XPC* (23, 61). For the differential expression analysis of selected radiation-induced genes, a two- factor ANOVA with interaction and Tukey’s honestly significant difference (HSD) test for post-hoc correction was performed on either individual or the pool of these six genes.

**Figure 2:**
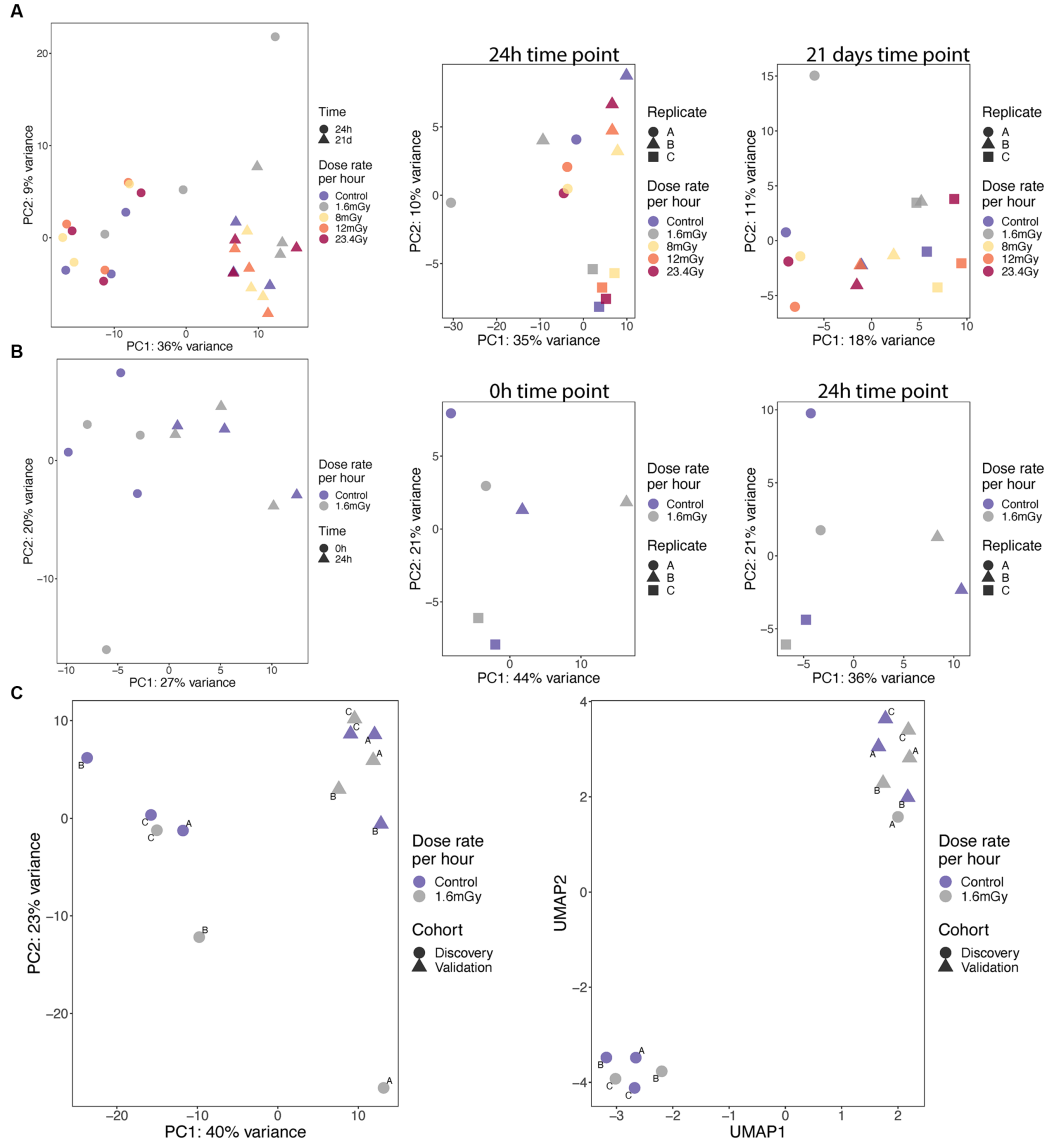
PCA analyses and correlation of expression on samples from discovery **(A)** and validation **(B)** cohorts at specified time points, as well as from pooled cohorts at 24 h (**C**, left), for which UMAP analysis was also performed (**C**, right).

Gene set enrichment analysis was done using the fgsea software (62) on KEGG pathways collection (63). Computation of signal transduction along signalling pathways was additionally performed using HIPATHIA (64) on KEGG pathways, after data normalization using box-cox transformation, *z*-score transformation and scaling from zero to one. Pathway level activation and functional level activation were obtained for each sample. Principal component analysis (PCA) was run for visualization of global differences between samples at pathway and functional level activation. Statistically significant activated pathways or functions were determined using the Limma test (65), considering each irradiated sample compared to the control at the same time point or each higher dose rate compared to each lower dose rate for a given time point.

Relative expression of *DMXL2* by RT-qPCR was analysed with GraphPad Prism, version 9, and statistical comparison between controls and 100 mGy-exposed cells (dose rate 1.6 mGy/h) was performed using an unpaired Student’s *t*-test. Data represent mean ± standard deviation from 3 independent experiments.

Cell growth curves were fitted to a second-order polynomial equation: $Y=B_{0}+B_{1}X+B_{2}X^{2}$ . One-way ANOVA was carried out on the B1 coefficient of cell growth curves and multiple comparisons were corrected using Tukey’s *post hoc* test. Absorbance and relative fluorescence units from cell viability assays were fit to a linear equation. Survival curves were fit to a linear quadratic equation $S=e^{-\left(\mathrm{aD}+\beta D^{2}\right)}$ , where *α* is the fitting coefficient and *D* is the total absorbed radiation dose in Gy. Data fitting was carried out with GraphPad Prism, version 5.

## Results

### Quality control of RNA-seq expression data

The number of reads per sample ranged from 24 to 42 million in the discovery cohort, and from 51 to 75 million in the validation cohort, with similar GC content (%) in all samples. Phred scores above 30 indicated good base quality at all positions for all samples. Illumina adapter content was similar between samples, and ~1% reads were removed by Trimmomatic in both cohorts. STAR alignment scores to human genome hg38 revealed 80 to 90% and 85 to 90% uniquely mapped reads in the discovery and validation cohorts, respectively. Only 2 to 3% of the reads were multimapped to different loci in both libraries. Almost all reads were mapped to protein-coding genes. Considering the limited number of gene expression changes that were ultimately validated at 24 h after 1.6 mGy/h, most of our observations regarding the discovery cohort alone will be included in the Supplementary material to give more weight instead to the validated and pooled cohort in the main figures.

### Global differences in gene expression between samples

PCA analyses were conducted using the 50% of most variable genes. PCA indicated no global differences between dose rates. High dependence on time point, with replicates clustering together according to either 24 h or 21 days, was observed in the discovery cohort (Figure 2A). A high variance between the replicates for a given dose rate was observed, as exemplified by the large variability between replicates A and B of the 1.6 mGy/h samples collected at 24 h after exposure in the discovery cohort (Figure 2A). No global differences between dose rates and high variance between replicates within the dose rates also applied to the validation cohort results (Figure 2B). Nevertheless, in the pooled cohort, the three replicates within each cohort clustered together. Discovery and validation cohorts presented significant differences between them, indicating a batch effect, as illustrated based on PCA and Uniform Manifold Approximation and Projection (UMAP) plots (Figure 2C). Generally, this indicates that radiation at LDLDR has very small global effects on gene expression within this cell type at the tested conditions. To test whether similar results would be obtained conducting our PCA analysis on the group of genes that showed significant expression changes relative to control cells, we firstly plotted PCA (Supplementary Figure S1A) and UMAP (Supplementary Figure S1B) having selected 389 genes with value of *p* < 0.05 (no correction for multiple testing) from a test comparing 1.6 mGy/h to control cells at 24 h in pooled data (discovery + validation). Results indicated a good separation of controls and irradiated samples, but the batch effect was still present. Secondly, we selected only 8 genes with value of *p* < 0.001, Supplementary Figures S1C,D, for PCA and UMAP, respectively. This second analysis led to discovery and validation cohorts starting to approximate each other, indicating that the batch effect decreased when very conservative DEGs *p*-value thresholds were selected.

**Figure 3:**
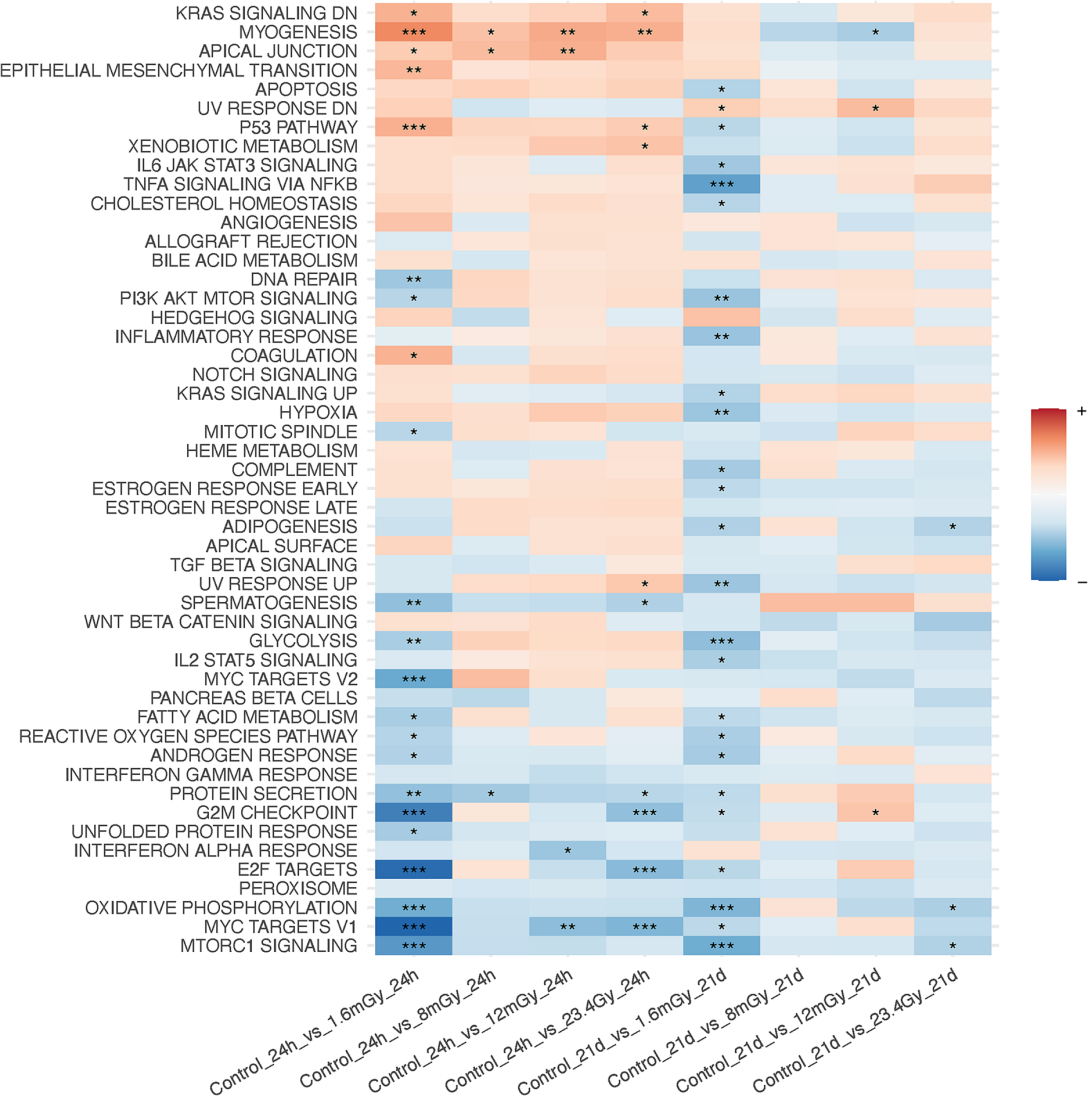
Gene set enrichment analysis on MSigDB hallmark pathways after data filtration on each irradiated sample as compared to the control of the same time point.

### Discovery of genes with expression changes between doses and time points

To increase the power of detection of differentially expressed genes in the discovery cohort, a GaMRed six components model was used to define a filtering threshold of expression to filter out low expressed genes. From the original 20,124 genes, a total of 8,410 low expression genes were filtered away according to a threshold equal to 8 of normalized expression data, so 11,714 genes remained for further analysis. Filtration resulted in formerly detected non-protein coding genes been excluded, leading to cleaner data. After filtration, 1,044 genes were upregulated and 866 downregulated at 1.6 mGy/h as compared to control at 24 h (Supplementary Figure S2A). Two genes were differentially expressed at 24 h as compared to control at 12 mGy/h, i.e., *ACTR10* (downregulated) and *C15orf59* (upregulated), none were altered at 8 mGy/h or 23.4 Gy/h (Supplementary Figure S2). At 21 days post-exposure, DEGs were only downregulated as compared to control, *KIF3C* and *VPS33B* at 1.6 mGy/h, *ENSG00000256204* at 8 mGy/h, and *LINC01119* and *USP41* at 23.4 Gy/h (Supplementary Figure S3). Comparisons of each higher dose rate to each lower dose rate led to the upregulation of 604 genes and the downregulation of 736 genes at 8 mGy/h as compared to 1.6 mGy/h at 24 h post-exposure. At 24 h, *FAM24B* was upregulated at 23.4 Gy/h compared to 12 mGy/h (Supplementary Figure S4). At 21 days after exposure, *ENSG00000256204* was upregulated at 12 mGy/h as compared to 8 mGy/h (Supplementary Figure S4), constituting the only DEGs found in these comparisons. At 23.4 Gy/h as compared to 12 mGy/h, the upregulation of *ABCA7* and downregulation of *USP41* were the only changes detected on day 21 (Supplementary Figure S4).

To identify genes with opposite responses at each dose rate relative to control between the 24 h and the 21 days time points, a two-factor analysis with DESeq2 for dose rate-time interactions was performed. Only single genes were found significant. For example, *VPS33B* was upregulated at 24 h, but downregulated 21 days after 1.6 mGy/h exposure as compared to control (Supplementary Figures S5A,E). Also, the expression of *HIST2H2AA4* showed opposite patterns for the higher dose rates as compared to control between the two time points (Supplementary Figures S5B–D,F). Concerning the panel of radiation-responsive genes from the literature, time was a statistically significant factor (Pr (>F) < 0.05) in the case of *BBC3*, *CDKN1A*, *GADD45A*, and *MDM2*, as well as for pooled genes. An increased passage number of these primary cells induced an elevated baseline level at 21 days compared to 24 h. No significant radiation-induced changes were noted at any dose rate.

GSEA performed after data filtration (Figure 3) showed that the KRAS signaling, apical junction, epithelial-mesenchymal transition, and the p53 pathways were upregulated in irradiated samples as compared to the control at the 24 h time point. Conversely, MTORC signaling, MYC targets V1 and V2, E2F targets, oxidative phosphorylation and the G2M checkpoint were downregulated. Moreover, when comparing each higher dose rate to the nearest lower one for a given time point, other than the 1.6 mGy/h comparison to the control already discussed, GSEA indicated that the strongest pathway alterations occurred at 24 h post exposure when comparing 8 mGy/h samples to those cells exposed at 1.6 mGy/h, Supplementary Figure S6. The latter revealed an upregulation of glycolysis, MYC targets V1 and V2, G2M checkpoint, oxidative phosphorylation, DNA repair and E2F targets. On the other hand, the epithelial-mesenchymal transition and the p53 pathways were downregulated.

**Figure 4:**
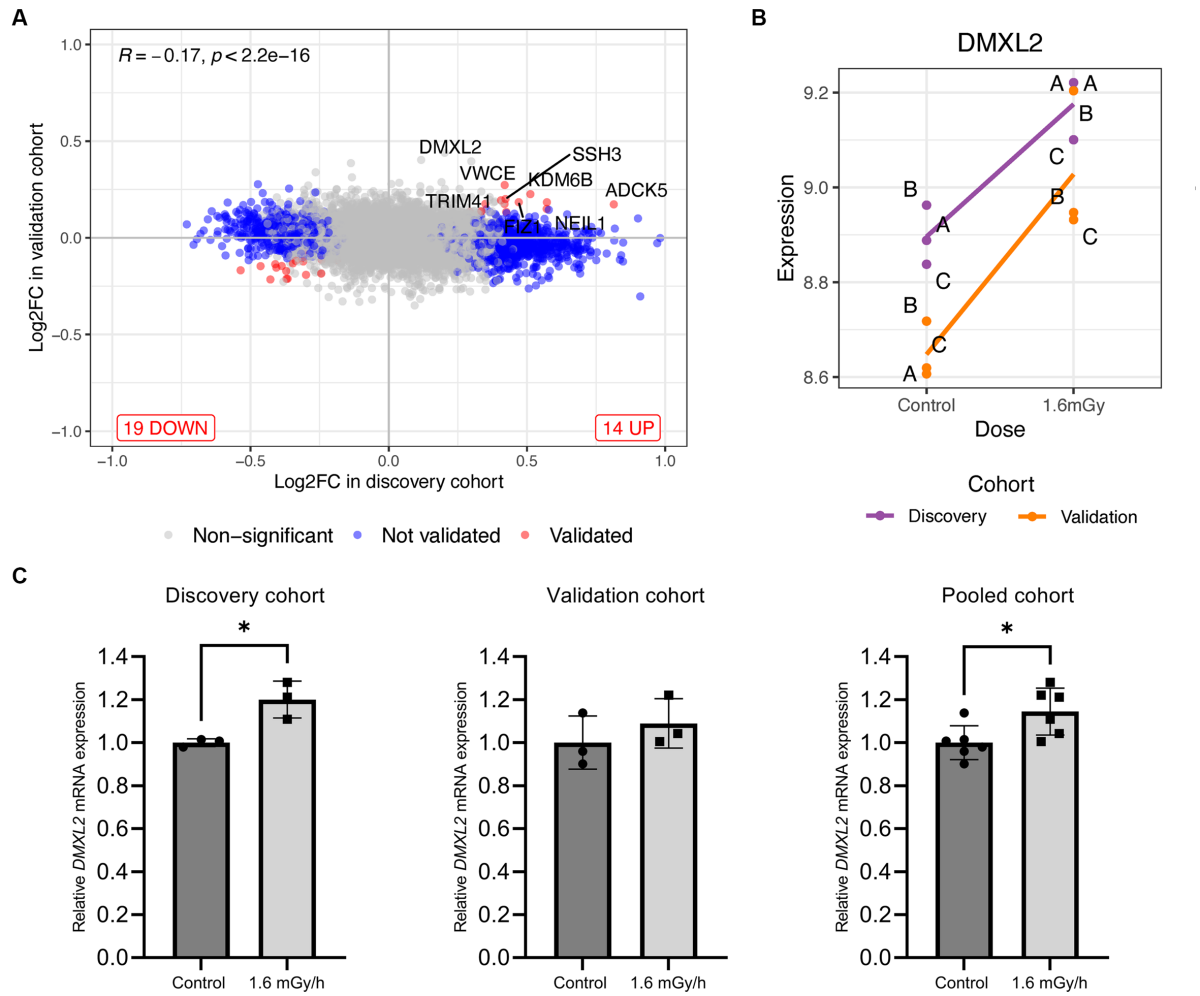
**(A)** Log2 fold change level comparison of differentially expressed genes in VH10 cells exposed to 1.6 mGy/h as compared to control at 24 h post exposure in discovery versus validation cohorts. Genes are classified by color according to their validation status, i.e., not validated and validated genes are shown in blue and red, respectively, while non-significant genes are depicted in grey. **(B)** Level of expression (log2 scale) of *DMXL2* in 1.6 mGy/h and control samples from discovery (purple) and validation (orange) cohorts, where **(A-C)** indicate biological replicates within each cohort. **(C)** *DMXL2* mRNA expression 24 h post exposure in control (dark grey) and 1.6 mGy/h (light grey) samples validated by qPCR in the discovery (*n* = 3; left), validation (*n* = 3, center), and pooled (*n* = 6, right) cohorts, respectively. Data is normalized to *18S* and expressed relative to average control values for each dataset.

At the level of KEGG pathway activation, 19 were up- and 10 downregulated at 24 h post-1.6 mGy/h exposure as compared to control. One pathway was downregulated at 8 mGy/h as compared to control and another one upregulated at 12 mGy/h as compared to control at 24 h. At 21 days after exposure, pathway level activation was only affected in samples exposed at 12 mGy/h as compared to control, i.e., 28 pathways were upregulated and one pathway was downregulated (Supplementary Figure S7). At the function level, it was the 1.6 mGy/h samples that differed relative to the control, with 17 up- and 4 downregulated functions, while no other conditions presented significant changes as compared to control (Supplementary Figure S8). Gene set enrichment analysis results on genes that presented differential responses between time points after data filtration are shown in Supplementary Figure S9. Here, the interferon alpha response, among others, was elevated at 21 days as compared to 24 h in 1.6 mGy/h samples, while the p53 pathway, oxidative phosphorylation, WNT/beta catenin and hedgehog signaling were downregulated.

### Validation of significant genes and pathways in an independent cohort

In contrast to the discovery cohort, only a few genes were significantly up- or downregulated in irradiated samples as compared to control. Just after exposure, *ENSG00000284691* was upregulated as compared to control, while *H4-16* was downregulated (Supplementary Figure S10A). At 24 h post-exposure, *ZC4H2* was the only transcript upregulated at 1.6 mGy/h as compared to control (Supplementary Figure S10B). Gene set enrichment analysis revealed just a few significant pathways activated in this cohort, either considering each irradiated sample to its corresponding control or dose rate-time interactions (Supplementary Figure S11).

No significant gene expression changes were detected for the panel of six radiation genes relative to control at any time point (Supplementary Figure S12), and contrary to the discovery cohort, where baseline levels at 21 days and 24 h differed, the time point (24 h versus 0 h) was not a significant factor in the validation cohort.

### Comparison of results between discovery and validation cohorts

Few genes showed consistent results across discovery and validation cohorts, meaning the same trend direction of differential expression at 1.6 mGy/h as compared to control at 24 h post-exposure. One such gene was *ADCK5*, which in both cohorts presented a trend towards upregulation (Supplementary Figure S13). Otherwise, the overall trend was towards lack of consistency, both at the gene and at the pathway level comparison. For the latter, only the apical junction pathway was found with a statistically significant activation 24 h post-exposure (Supplementary Figure S14).

### Gene expression analysis using pooled cohort

**Figure 5:**
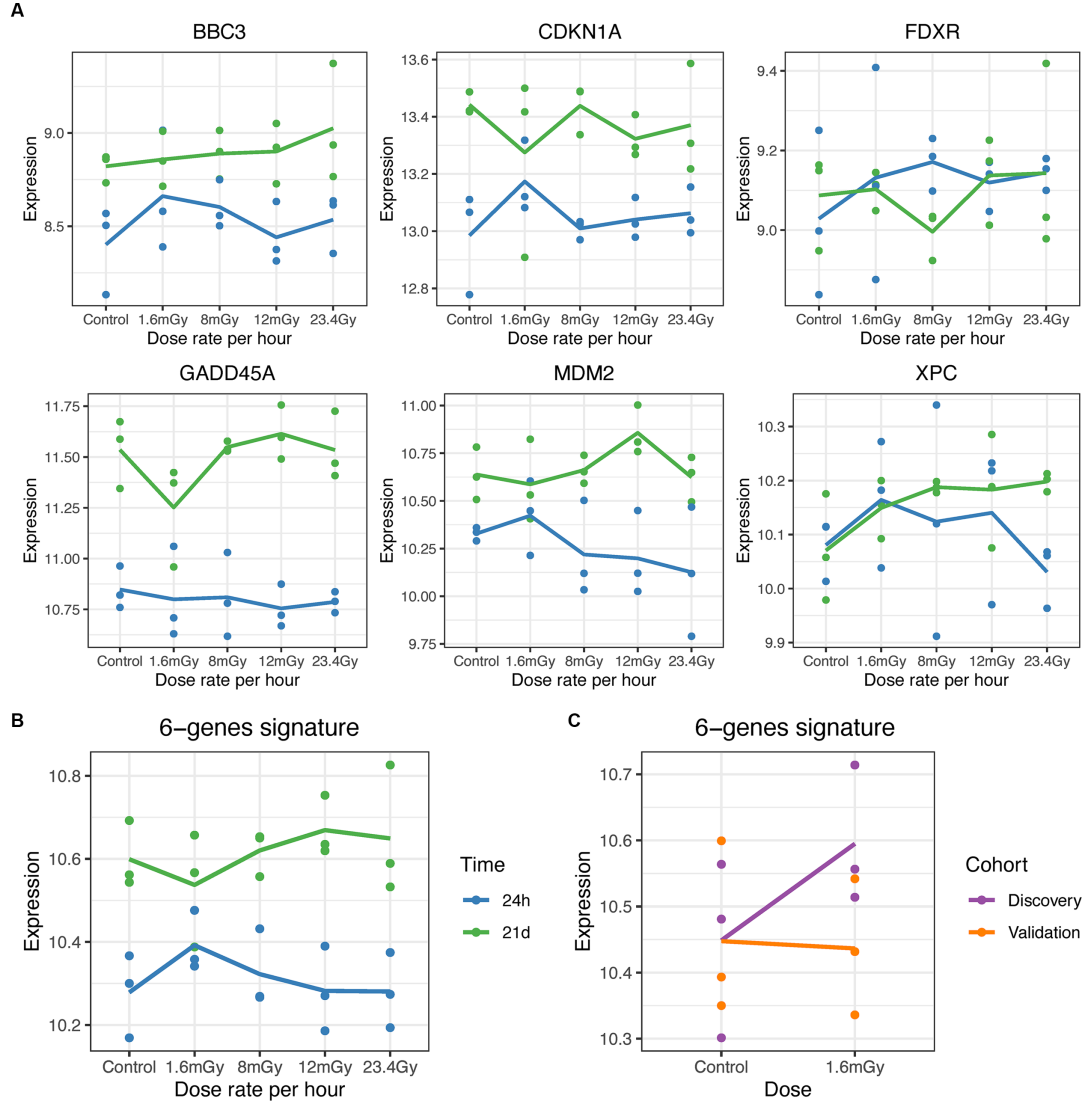
Gene expression level (log2 scale) of known radiation-induced genes. **(A)** Gene expression of *BBC3*, *CDKN1A*, *FDXR*, *GADD45A*, *MDM2*, and *XPC* at 24 h (blue) and 21 days (green) post exposure at the different dose rates. **(B)** Expression changes in the pool of genes in cells exposed at 1.6 mGy/h as compared to control at 0 and 24 h post exposure. **(C)** Expression changes in the pool of genes in cells exposed at 1.6 mGy/h as compared to control at 24 h post exposure in the discovery (purple) and validation (orange) cohorts.

**Figure 6:**
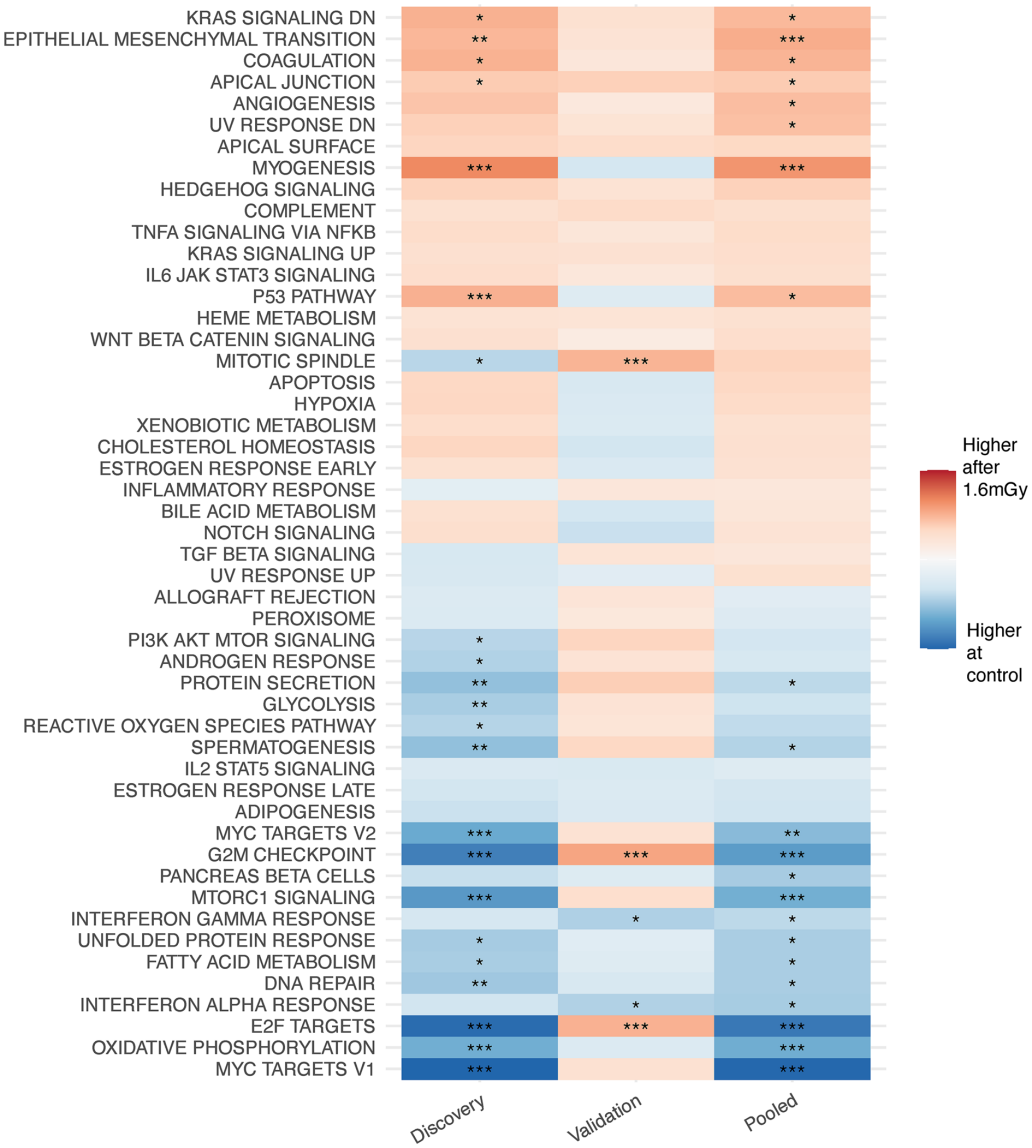
Gene set enrichment analysis on MSigDB hallmark pathways from discovery, validation and pooled data on VH10 exposed at 1.6 mGy/h as compared to control 24 h post exposure.

Univariate analysis on the pooled cohort, including cohort as a covariate to reduce the batch effect, resulted in a decrease of DEGs as compared to the discovery cohort. Figure 4A shows the volcano plot of DEGs in validation and discovery cohorts, whereby few genes were validated, i.e., those indicated in red, Table 1. Among these, only *DMXL2* was indeed significantly upregulated in the pooled cohort. Both the discovery and validation cohorts presented the same upregulation trend of *DMXL2*, yet with a pattern of lower magnitude of response in the discovery cohort (Figure 4B). Statistically significant upregulation of *DMXL2* in the discovery cohort was subsequently confirmed by RT-qPCR, while no significant difference in expression level between the control and 1.6 mGy/h-exposed cells was observed in validation cohort (Figure 4C left and center, respectively). In the pooled cohort, the expression level in exposed cells was significantly increased (Figure 4C, right). Overall, the magnitudes of fold changes of *DMXL2* were relatively small in the discovery and pooled cohorts from both RNA sequencing and RT-qPCR data (Table 1 and Figure 4B). Moreover, no significant differences relative to control were detected concerning the level of expression of the panel of six radiation responsive genes after the different dose rates and time points (Figure 5), for either each individual gene (Figure 5A) or the pool of genes (Figures 5B,C), whereby the originally observed pattern of upregulation at 1.6 mGy/h as compared to control in the discovery cohort could not be further validated (Figure 5C). Finally, gene set enrichment analysis on the pooled cohort showed similar findings as in the discovery cohort, revealing significantly up- and downregulated pathways, some of them also identified in the validation cohort. These included the EMT, p53 pathway and the DNA repair pathway to name a few examples (Figure 6).

**Table 1:** List of validated genes in VH10 cells exposed to 1.6 mGy/h as compared to control at 24 h post exposure corresponding to those presented in red in Figure 4A.

Gene	L2FC_disc	Pval_disc	Padj_disc	L2FC_val	Pval_val	Padj_val
*ADCK5*	0.8141	0.0000	0.0094	0.1728	0.1311	1
*ADAMTS9*	0.5722	0.0025	0.0604	0.1838	0.1248	1
*NEIL1*	0.5713	0.0031	0.0658	0.1494	0.1652	1
*KDM6B*	0.5123	0.0072	0.0971	0.2258	0.0391	1
*FIZ1*	0.4703	0.0046	0.0808	0.1842	0.0795	1
*TRIM41*	0.425	0.0016	0.0498	0.1335	0.1615	1
*SSH3*	0.4224	0.0122	0.1262	0.2006	0.0689	1
*VWCE*	0.4194	0.0260	0.1774	0.2729	0.0147	1
*PLXNB1*	0.4191	0.0199	0.1577	0.1749	0.1307	1
*CSNK1G2*	0.4061	0.0123	0.1263	0.1940	0.0700	1
*DMXL2*	0.2987	0.0781	0.3041	0.3956	0.0002	0.5666
*MRPS23*	−0.2992	0.0089	0.1082	−0.1918	0.0106	1
*MX1*	−0.3658	0.0212	0.1625	−0.2120	0.0640	1
*ARL6IP6*	−0.3699	0.0185	0.1525	−0.2125	0.0173	1
*METTL6*	−0.3707	0.0040	0.0751	−0.1721	0.0686	1
*SNRPD1*	−0.3872	0.0274	0.1819	−0.1462	0.1086	1
*SEC22A*	−0.4067	0.0065	0.0934	−0.1546	0.0901	1
*FIGNL1*	−0.4105	0.0236	0.1705	−0.1368	0.1903	1
*FLRT3*	−0.4281	0.0245	0.1729	−0.2158	0.0536	1
*ELOVL6*	−0.4636	0.0208	0.1609	−0.1468	0.1836	1
*CDC25C*	−0.5358	0.0113	0.1218	−0.1683	0.1232	1

Additionally, *DMXL2* is included in the list as it was the only significant gene in the pooled cohort. L2FC, Log2FoldChange; disc, discovery; val, validation; Pval, *p* value; Padj, *p* value adjusted.

### Low dose and low dose rate radiation have no cytotoxic effects on VH10 fibroblasts

**Figure 7:**
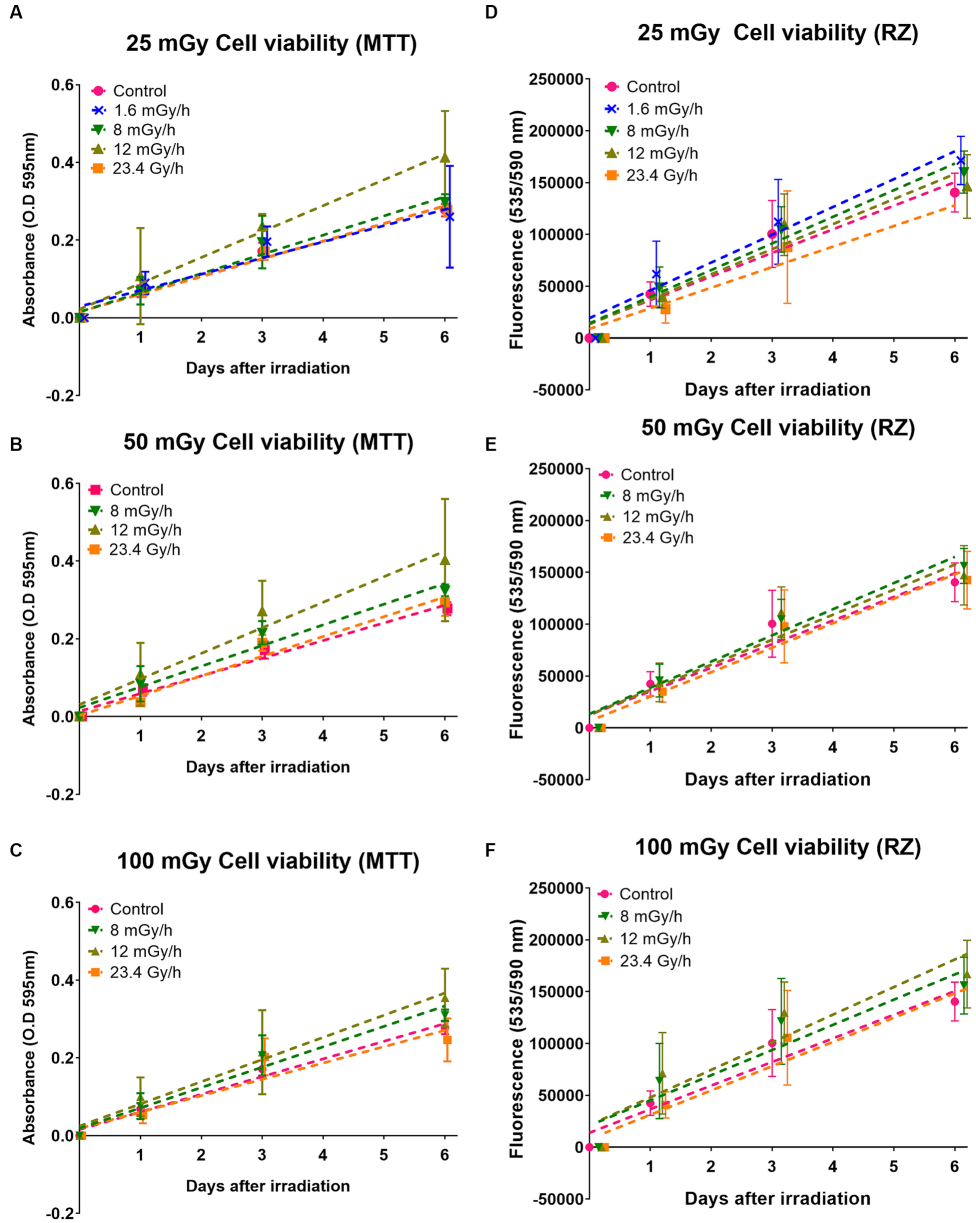
Cell viability with MTT and resazurin (RZ): effects of gamma radiation exposure at low dose rates at doses 25, 50, and 100 mGy on cell growth analysed with two different cell viability assays. **(A–C)** Represent cell growth determined with MTT assay, while **(D–F)** represent cell growth determined with resazurin (RZ) assay. Five independent experiments were conducted. Dashed lines represent fit lines (linear regression) at different dose rates. Note these values were analysed as a function of different dose rates at a particular dose.

To determine the potential cytotoxic effects of LDLDR exposure, we first investigated temporal changes in VH10 cell growth. Cell growth effects were categorized as either short-term, i.e., occurring from 24 h until 6 days after radiation exposure, or long-term effects, i.e., changes occurring weeks after radiation exposure. To determine short-term effects, two cell viability assays assaying the metabolic activity of cells, the MTT and the resazurin assays (absorbance or fluorescence output, respectively), were performed simultaneously 1, 3, and 6 days after exposure to doses of 25, 50, and 100 mGy gamma radiation at the dose rates of 1.6, 8, 12 mGy/h, and 23.4 Gy/h (Figure 7; Supplementary Figure S15). Two-way ANOVA analysis did not indicate any statistically significant change in cell viability either in comparison with non-irradiated control or between different irradiated groups. However, we observed a tendency for a slight increase in cell growth in cells exposed to 25 mGy at 1.6 mGy/h (Supplementary Figures S15A,E). For the lowest dose rate, i.e., 1.6 mGy/h, the cell viability assay was only performed after 25 mGy (15 h exposure time) since the longer exposure times required to achieve higher doses would make comparisons between dose rates difficult at the selected time points. A slight increase in cell growth of cells exposed to 12 mGy/h at all doses was also observed with the MTT assay but not the resazurin reduction assay (Figure 7). We reanalyzed our data as a function of dose at each dose rate (Supplementary Figure S15) and observed inconsistent trends between both assays at all doses except 100 mGy. Although also statistically insignificant, VH10 fibroblasts irradiated at 100 mGy showed a tendency for a slight increase in cell growth in cells exposed at dose rates of 12 and 8 mGy/h.

**Figure 8:**
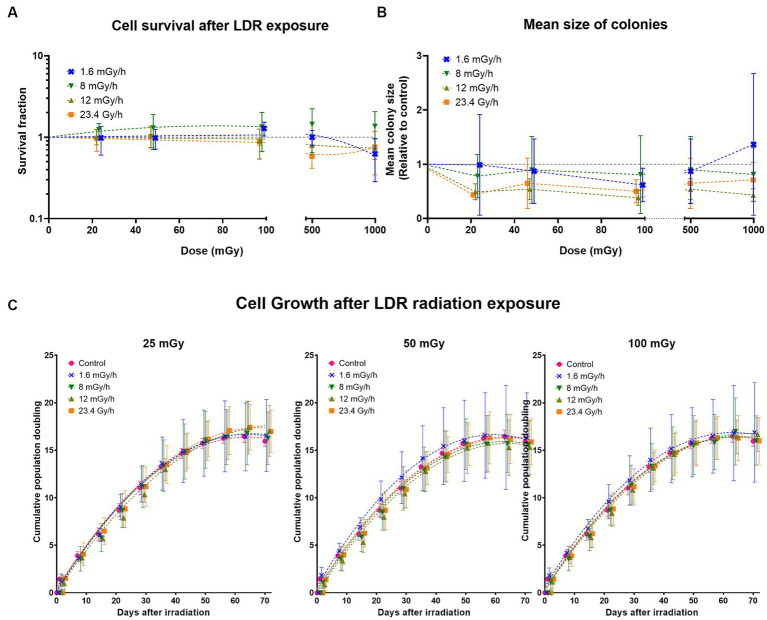
Long-term effects of gamma radiation at low dose rates (LDR) 1.6, 8, 12 mGy/h and high dose rate 23.4 Gy/h. **(A)** Cell survival after LDLDR exposure. The dashed colored lines represent fits to a linear quadratic equation. The missing line in 8 mGy/h is due to the large uncertainties at this dose rate which drives the fits down the survival fraction range represented on the *Y*-axis. **(B)** Mean size of colonies. The dashed colored lines represent connecting lines, while the dashed horizontal grey line represents the non-irradiated control. **(C)** Cumulative population doublings at given doses. Five independent experiments were conducted with growth curves of cells irradiated at different dose rates at doses 25, 50, and 100 mGy. Data was fit using a second order polynomial equation. Dashed lines represent fit lines at different doses and dose rates.

The discrepancies observed between the MTT and resazurin cell viability assays led to performing colony forming assay to determine the effect on cell survival (colony number) and proliferation (colony size). Cell survival at low dose rates 1.6, 8, and 12 mGy/h was compared to cell survival at 23.4 Gy/h. At doses ≤100 mGy, no difference in cell survival was observed either between dose rates or compared to the non-irradiated control whose surviving fraction is 1 (Figure 8A). However, at doses >100 mGy a slight reduction in surviving fraction was present at all dose rates (not statistically significant), except 8 mGy/h which tended to increase. Surviving fraction is calculated as a function of the number of colonies counted. In comparison to the non-irradiated control, our results indicate no statistically significant difference in the number of colonies at any dose or dose rate. We analysed colony size as a parameter for cell proliferation, presented as relative values to the control (Figure 8B). One-way ANOVA analysis comparing colony size at each dose to the control indicates a decline in colony size at all doses below 100 mGy in cells irradiated at both 23.4 Gy/h and 12 mGy/h (not statistically different at 50 mGy). The pattern was similar at doses above 100 mGy, but a statistically significant decrease was only observed in cells irradiated at 12 mGy/h, not 23.4 Gy/h. We observed no statistically significant differences relative to the control at either 1.6 or 8 mGy/h, but a tendency towards a decrease was present at 8 mGy/h as well.

To determine the latent effects of radiation exposure at LDLDR on cell growth, if any, VH10 cells were passaged weekly for 70 days (10 weeks) after 25, 50, and 100 mGy exposure at dose rates of 1.6, 8, 12 mGy/h, and 23.4 Gy/h. We did not observe any statistically significant changes in long-term cell growth patterns at any dose or dose rate (Figure 8C; Supplementary Figure S16).

## Discussion

Resolving the shape of dose response relationship at low doses and dose rates requires highly radiosensitive biomarkers and a better understanding of the biological response under these conditions. There is an ongoing search within the field for radiosensitive biomarkers using numerous endpoints (14, 16). Here, we investigated LDLDR-driven short and long-term changes in gene expression (100 mGy), viability and survival (25–100 mGy), as well as long-term effects on cell growth in VH10 fibroblasts exposed at different dose rates. The 100 mGy dose is clinically relevant as it would be equivalent to that absorbed by healthy tissue in out-of-field beam during a single radiotherapy fraction (45). Cell-based studies on the effect of LDLDR on cell growth and cell survival provide a context to analyze our transcriptomic results, which serve as a readout of early and late cellular responses to radiation exposure.

RNA-seq results from the discovery cohort showed the up- and downregulation of several genes at 24 h post-exposure to 1.6 mGy/h as compared to control in VH10 cells. However, a latter validation cohort, and the pooled data from these, did not support the majority of those gene-specific findings. Pooling and normalizing the data of both cohorts together improved the statistical power and contributed to better control of the impact of average versus variance and the fact that the number of sequencing reads was increased in the validation cohort as compared to the discovery. Based on the pooled data, no strong gene expression changes were observed in chronically exposed VH10 cells. The small effects on gene expression in human fibroblasts, i.e., less than two-fold as compared to control, following low dose exposure are in agreement with previous findings (28, 36). As opposed to high doses (1–5 Gy), primary human fibroblasts exposed to 100 mGy ^137^Cs gamma rays at a dose rate of 2.82 Gy/h (47 mGy/min) did not show differential gene expression as compared to unirradiated cells 4 h after sham- or IR-exposure (36). Moreover, gene expression changes were transient and returned to baseline at 24 h post-exposure in skin biopsies (30). Sokolov et al. concluded that a 2-fold cut-off rejected 80–83% of DEGs following 1 Gy gamma exposure in human fibroblasts (66). Thus, gene expression effects are small even at a moderately high dose of 1 Gy in this cell type. Nevertheless, low dose-driven alterations on transcriptional (28, 29) or cell cycle regulation genes have been described by others (29, 32). In our results, only the *Dmx-like 2* (*DMXL2*) gene presented a moderate but consistent upregulation trend among cohorts. The upregulation of this gene was further validated by qPCR.

*DMXL2* (67), named after its first homolog identified on the X chromosome of *Drosophila melanogaster* (68), is located on chromosome 15q21.2 and encodes a 12-WD domain (repeat motif, terminating in a tryptophan-aspartic acid (W-D) dipeptide) DmX-like protein 2, also known as rabconnectin-3α (Rbcn-3α) (69). DMXL2 is involved in the regulation of the NOTCH signalling pathway in mammalian cells (70), through V-ATPase complex-driven acidification of endocytic compartments (70, 71). A global decrease in ATP levels was observed in human fibroblasts exposed to low doses delivered at intermediate dose rates, while the same dose delivered acutely, lead to an increase in ATP, hypothesized to drive survival differences (72). This would be in line with others, who propose the ADP:ATP ratio as an indicator of cell viability, necrosis and apoptosis (73). As being ubiquitously expressed (74), *DMXL2* is associated with several conditions (75–83) and is regarded as a functional biomarker of estrogen receptor alpha (Erα) positive breast cancer (71). *DMXL2* drives NOTCH signalling and the mesenchymal switch in endocrine therapy-resistant breast cancer cells when overexpressed (71). Interestingly, both NOTCH and EMT signalling pathways were represented in our GSEA results. In the discovery and in the pooled data cohort, the NOTCH signalling pathway was upregulated (not statistically significant), while it was downregulated in the validation cohort (not statistically significant). An enrichment with a positive correlation of the NOTCH signalling pathway is observed in human primary fibroblasts firstly primed with a low dose of 50 mGy and later challenged with 2 Gy of X-rays, but not in the low or high-exposed cells only (32). EMT is activated by several signalling pathways, including NOTCH (84). The EMT pathway was upregulated in all cohorts, statistically significant both in the discovery and in the pooled data cohort 24 h post-exposure in VH10 cells exposed to 100 mGy at 1.6 mGy/h as compared to control. EMT induction leads to the acquisition of stem-cell properties in human epithelial cells (85, 86). A loss of epithelial markers, such as E-cadherin and tight junction proteins, is associated with EMT (87), a key step in cancer progression (88). Additionally, some genes presented a similar profile in both the discovery and the validation sets, such as *ADCK5*, which presented a relatively consistent dose rate-independent radiation-induced pattern as well, and has previously been found to have a role in invasion and migration in lung cancer cells (89).

Overall, few consistent changes were observed at the pathway level. These included: a consistent upregulation of the apical junction pathway and a downregulation of the DNA repair pathway in all three cohorts (statistically significant in discovery and pooled cohorts). The apical junction complex comprises adherent junctions, formed by E-cadherin, and tight junctions, and altogether acts as a hub of signal transduction system at the lateral membrane of epithelial cells to regulate cell–cell adhesion, transcription, proliferation and differentiation (90). Lastly, *DMXL2* isoform 3, upregulated in HER2+/ER−/PR− breast cancers, was computationally predicted to be associated with glycolysis (91, 92). Glycolysis was downregulated both in the discovery (statistically significant) and in the pooled data cohort but was non-statistically upregulated in the validation cohort. Again, small effects at the pathway level were in agreement with previous studies, where significant changes could not be detected in either primary human fibroblasts (31) or *in vivo* (30) following low doses of IR.

The tumor suppressor gene *TP53* is a key regulator of radiation-induced gene expression changes (93, 94). The p53 pathway was statistically significantly upregulated in the discovery and pooled data cohorts, but non-significantly downregulated according to the validation cohort results. Moreover, we did not detect gene expression changes in the panel of *BBC3*, *CDKN1A*, *FDXR*, *GADD45A*, *MDM2*, and *XPC*, which are six well-known p53-target radiation-responsive genes (23, 61). However, we have observed a ca 2-fold upregulation of *CDKN1A* and *MDM2* at 4 h after 0.2 Gy and 3-fold upregulation following 1 Gy X-ray exposure in VH10 (manuscript in preparation), indicating that in these cells a DNA damage response is triggered by moderate to high doses of IR. It is known that *TP53* plays a dominant role at high doses (29, 36, 39). In agreement, Ding et al. only observed induction of *MDM2* and *CDKN1A* following high but not low doses in normal human fibroblasts (28). A dose-rate-dependent induction of *CDKN1A* and *GADD45A* genes and other apoptosis-related genes occurs following low dose exposure in the human myeloid leukemia ML-1 cell line (48). It is then plausible that low doses and dose rates of IR led to a p53 (or this panel of genes)-independent response in VH10 cells, that the time point of choice did no capture their contribution, or perhaps most likely in the context of the limited overall gene expression response, that such low response in this relatively resistant cell type was just below the detection limit. Nevertheless, others have reported p53 signalling pathway activation at 3 h post-exposure to 100 mGy delivered at 63 mGy/min using a dermis model, and a small upregulation (FC < 1.5) of the *GADD45*, *MDM2*, and *CDKN1A* genes was observed, probably indicative of IR-induced cell cycle arrest (45). The same dose of 100 mGy delivered at 0.5 Gy/min led to gene expression changes indicative of G2/M accumulation at 24 h post-exposure in a human 3D skin model (35). Here, the G2/M checkpoint was statistically significantly downregulated in the discovery and pooled data cohorts, but significantly upregulated in the validation cohort. Previously, it has been suggested that a certain quantity of DNA double-strand breaks might determine whether G2/M cell cycle arrest is, or not, induced (95). Despite the evidence of the occurrence of DNA damage at doses as low as approximately 1 mGy, there is also evidence that below a certain threshold, cells are incapable of recognizing and signalling DNA double-strand breaks (DSBs) (96). Abrogation of the early response to LDLDR has also been described (97). If cells are incapable of DNA damage sensing or response below a certain dose threshold, then aberrant DNA damage repair should serve as endpoints, but these markers usually entail high uncertainties at low doses (16).

The interpretation of transcriptomic data at a specific time point after exposure, although informative, is rather incomplete without a comprehensive follow-up and integration with other parameters, such as other omics (10) or survival (98) data. Transcriptional studies on IR-exposed fibroblasts have typically focused on early responses only, i.e., <72 h after exposure (10, 28, 39), leaving sustained long-term changes unexplored. Endpoints such as survival (72, 99, 100), γ-H2AX foci, apoptosis or ATP level (72) have been studied following different dose rates in human fibroblasts. As compared to control, Dionet et al. did not observe any significant difference in survival in normal K464 human fibroblasts exposed to 50, 100, and 150 mGy neutrons with an average energy of 33 MeV at a medium dose rate, i.e., 64.8 mGy/h (1.08 mGy/min) (72). However, survival was significantly diminished at 250 mGy, indicating a threshold of approximately 150 mGy above which the efficient repair of IR-induced lesions starts to be compromised at lower dose rates (72). Conversely, at the intermediate rate of 83 mGy/min, the survival of K464 fibroblasts decreased with the dose and was already significantly different from control cells at 100 mGy neutron dose, without an apparent threshold of dose (72). At doses up to 50 Gy, Nagasawa et al. also observed an increase in the inverse of the slope (D_0_) in the survival curves of six normal human skin fibroblast strains when irradiated at continuous low dose rate (0.023 or 0.153 Gy/h) as compared to acute high dose rate (0.70 to 0.75 Gy/min) gamma rays (99). These results indicate a higher effectiveness of low doses when delivered at high dose rates in normal human fibroblasts at the level of survival. Given that γ-H2AX foci levels were comparable between lower (1.08 mGy/min) and higher (83 mGy/min) neutron dose rates 24 h after exposure, the described dose rate effect at the survival level was not suggested to be explained by differences in the signalling of double strands breaks, nor by apoptosis as determined by active caspase-3 fluorescence expression at 24 h after exposure (72).

Cell viability assays, the colony forming assay, and an exhaustive follow-up of cell growth represented a feasible approach to analyze the cytotoxic effects of low doses and dose rates to complement our gene expression data. Here, a tendency for increased cell proliferation was observed. Similar studies have shown that LDLDR exposure stimulates cell proliferation via the AKT and ERK signalling pathways in normal human lung fibroblasts (101, 102). This fits to our early response assays (1–7 days after exposure) but is contrary to our data presented in Figure 8 on colony size, which does not indicate increased cell proliferation within the surviving colonies at 14 days after exposure. Besides, the AKT signalling pathway was downregulated in the discovery and pooled data cohorts and upregulated in the validation cohort. No significant changes in proliferation relative to control were found in the dermis of a skin model 24 h after exposure to 100 mGy X-rays, although a non-statistically significant trend of increased proliferation was observed at 72 h (45). The lack of statistically significant data coupled with the discrepancies observed from our cell viability data complicate conclusions of the short-term effect that LDLDR exposure has on VH10 fibroblasts. One further limitation was the variability observed among the controls in the RNA-seq cohorts. A plausible explanation for the observed variability could be the impact of uncontrollable factors, such as environmental conditions or the physiological state of the cells. As compared to high doses, the variability at low doses is expected to be larger considering the more parameters involved (103), which although also present in high dose experiments, are likely to be hidden by stronger effects. On a chronic exposure experimental setup, involving low doses and dose rates, if anything, exposure at these conditions is essentially likely to induce small effects. Moreover, because of our chronic exposure setup, fibroblasts were not synchronized unlike in previous studies (28, 31, 36, 39), so cell cycle-dependent effects may have additionally contributed to the observed, and to some extent expected, variability.

Skin has a relatively low radiation detriment value and tissue weighting factor, which as defined by the ICRP, reflect the relative contribution of a specific organ or tissue to the risk of radiation-induced stochastic effects. This is related to the low severity of basal cell carcinoma induced by radiation and does not exclude the possibility of effects at <100 mGy, specially under *in vitro* experimental conditions in one of the cell types present in skin, i.e., fibroblasts. However, our results suggest that low doses of IR do not lead to substantial cellular effects in VH10. Except for a moderate trend of upregulation of the *DMXL2* gene, no strong consistent gene expression changes were detected in VH10 cells after 100 mGy delivered at different low dose rates and a single acute dose rate at either early or late time points post-exposure. GSEA indicated downregulation of DNA repair pathways, which could potentially translate into cytotoxic effects. However, cell viability assays and agarose overlay colony forming assays did not support any statistically significant changes in cell growth or survival. At 12 mGy/h and 23.4 Gy/min, tendencies of increased growth and reduced colony size were noticed as compared to control. Finally, a 70 days follow-up on cell growth did not reveal significant differences relative to control at any dose rate or dose. Increasing evidence indicates that cells exposed to LDLDR may exhibit a different biological response as compared to high doses and dose rates (104). Concerning the dose rate effect, DDREF is likely to vary depending on the dose, radiation quality, cell type and endpoint (105, 106). Although here low doses and low dose rates of gamma radiation had no cytotoxic effects on VH10 cells, the results should be interpreted with caution. They in no way contradict the linear no-threshold (LNT) model adopted by the ICRP for radiation protection, which assumes that the biological response of cells exposed to low doses varies only in magnitude as compared to high doses (107).

Our transcriptomic results revealed *DMXL2* as the unique differentially expressed gene as compared to control. *DMXL2* had a consistent trend of moderate upregulation at 24 h post-exposure to 100 mGy at 1.6 mGy/h in the three cohorts analysed (discovery, validation and pooled). The NOTCH signalling pathway, partially regulated by *DMXL2*, was upregulated (not statistically significant) in two of the cohorts according to gene set enrichment analysis (GSEA). Other than weak tendencies of increased growth and reduced colony size at 12 mGy/h and 23.4 Gy/min, no statistically significant early effects in cell viability or survival patterns were detected. Besides, no long-term effect on cell growth was identified. Altogether, these results indicate weak or undetectable effects of low doses and low dose rates of ionizing radiation in VH10 fibroblasts under the studied conditions.

## Data availability statement

The datasets presented in this study can be found in online repositories. The names of the repository/repositories and accession number(s) can be found at: www.ncbi.nlm.nih.gov/sra/PRJNA1019267, SRA.

## Ethics statement

Ethical approval was not required for the studies on humans in accordance with the local legislation and institutional requirements because only commercially available established cell lines were used. Ethical approval was not required for the studies on animals in accordance with the local legislation and institutional requirements because only commercially available established cell lines were used.

## Author contributions

PA: Formal analysis, Methodology, Visualization, Writing – review & editing. ML-R: Visualization, Writing – original draft, Writing – review & editing. MM: Formal analysis, Visualization, Writing – review & editing. ZK: Methodology, Formal analysis, Writing-review & editing. FB: Methodology, Writing – review & editing. JP: Formal analysis, Writing – review & editing. AW: Conceptualization, Funding acquisition, Project administration, Supervision, Writing – review & editing. LL: Project administration, Supervision, Writing – review & editing.
