# Erratum: Short- and long-term effects of radiation exposure at low dose and low dose rate in normal human VH10 fibroblasts

**DOI:** 10.3389/fpubh.2024.1400355

**Published:** 2024-04-10

**Authors:** Frontiers Production Office

**Affiliations:** Frontiers Media SA, Lausanne, Switzerland

**Keywords:** low dose, low dose rate, dose and dose rate effectiveness factor, radiation carcinogenesis, fibroblasts

Due to a production error, the in-text citations did not refer to the correct Figures.

A correction has been made to the section **Materials and methods**, *Statistical analysis*, paragraph 1.

The sentence previously said:

“Regarding Figure 2, a two-factor ANOVA with interaction and Tukey's honestly significant difference (HSD) test for *post-hoc* correction on irradiated samples compared to the corresponding control of the same time point, was performed on either individual or the pool of these six genes.”

The correct sentence appears below:

“For the differential expression analysis of selected radiation-induced genes, a two-factor ANOVA with interaction and Tukey's honestly significant difference (HSD) test for post-hoc correction was performed on either individual or the pool of these six genes.”

Due to a production error, “Figure 2A” was incorrectly cited as “Figure 3A,” “Figure 2B” was incorrectly cited as “Figure 3B,” and “Figure 2C” was incorrectly cited as “Figure 3C” in the article. The citations have now been corrected in the section **Results**, *Global differences in gene expression between samples*. The corrected sentences within the paragraph appear below:

“High dependence on time point, with replicates clustering together according to either 24 h or 21 days, was observed in the discovery cohort (Figure 2A). A high variance between the replicates for a given dose rate was observed, as exemplified by the large variability between replicates A and B of the 1.6 mGy/h samples collected at 24 h after exposure in the discovery cohort (Figure 2A). No global differences between dose rates and high variance between replicates within the dose rates also applied to the validation cohort results (Figure 2B). Nevertheless, in the pooled cohort, the three replicates within each cohort clustered together. Discovery and validation cohorts presented significant differences between them, indicating a batch effect, as illustrated based on PCA and Uniform Manifold Approximation and Projection (UMAP) plots (Figure 2C).”

Due to a production error, “Figure 3” was incorrectly cited as “Figure 4” in the article. The citation has now been corrected in the section **Results**, *Discovery of genes with expression changes between doses and time points*, paragraph 3 and should read:

“GSEA performed after data filtration (Figure 3) showed that the KRAS signaling, apical junction, epithelial-mesenchymal transition, and the p53 pathways were upregulated in irradiated samples as compared to the control at the 24-h time point.”

Due to a production error, “Figure 4A” was incorrectly cited as “Figure 5A”, “Figure 4B” was incorrectly cited as “Figure 5B” and “Figure 4C” was incorrectly cited as “Figure 5C” in the article. “Figure 5” was incorrectly cited as “Figure 2”, “Figure 5A” was incorrectly cited as “Figure 2A”, “Figure 5B,C” was incorrectly cited as “Figure 2B,C” and “Figure 5C” was incorrectly cited as “Figure 2C” in the article.

The citations have now been corrected in the section **Results**, *Gene expression analysis using pooled cohort*, and should read:

“Univariate analysis on the pooled cohort, including cohort as a covariate to reduce the batch effect, resulted in a decrease of DEGs as compared to the discovery cohort. Figure 4A shows the volcano plot of DEGs in validation and discovery cohorts, whereby few genes were validated, i.e., those indicated in red, Table 1. Among these, only DMXL2 was indeed significantly upregulated in the pooled cohort. Both the discovery and validation cohorts presented the same upregulation trend of DMXL2, yet with a pattern of lower magnitude of response in the discovery cohort (Figure 4B). Statistically significant upregulation of DMXL2 in the discovery cohort was subsequently confirmed by RT-qPCR, while no significant difference in expression level between the control and 1.6 mGy/h-exposed cells was observed in validation cohort (Figure 4C left and center, respectively). In the pooled cohort, the expression level in exposed cells was significantly increased (Figure 4C, right). Overall, the magnitudes of fold changes of DMXL2 were relatively small in the discovery and pooled cohorts from both RNA sequencing and RT-qPCR data (Table 1 and Figure 4B). Moreover, no significant differences relative to control were detected concerning the level of expression of the panel of six radiation responsive genes after the different dose rates and time points (Figure 5), for either each individual gene (Figure 5A) or the pool of genes (Figures 5B,C), whereby the originally observed pattern of upregulation at 1.6 mGy/h as compared to control in the discovery cohort could not be further validated (Figure 5C). Finally, gene set enrichment analysis on the pooled cohort showed similar findings as in the discovery cohort, revealing significantly up- and downregulated pathways, some of them also identified in the validation cohort. These included the EMT, p53 pathway and the DNA repair pathway to name a few examples (Figure 6).”

The publisher apologizes for these mistakes. The original article has been updated.

