# Supplementary material for: Short- and long-term effects of radiation exposure at low dose and low dose rate in normal human VH10 fibroblasts
Source: Front Public Health. 2023 Dec 15;11:1297942. doi: 10.3389/fpubh.2023.1297942 (PMC10755029; doi:10.3389/fpubh.2023.1297942)
Supplement: Supplementary file 1 [file Image_1.pdf]

Supplementary Material

Short- and long-term effects of radiation exposure at low dose and low dose rate on normal human VH10 fibroblasts

Pamela Akuwudike, Milagrosa López-Riego*, Michal Marczyk, Zuzana Kocibalova, Fabian Brückner, Joanna Polańska, Andrzej Wojcik, Lovisa Lundholm

*** Correspondence:** Corresponding Author: milagrosa.lopezriego@su.se

Supplementary Figures

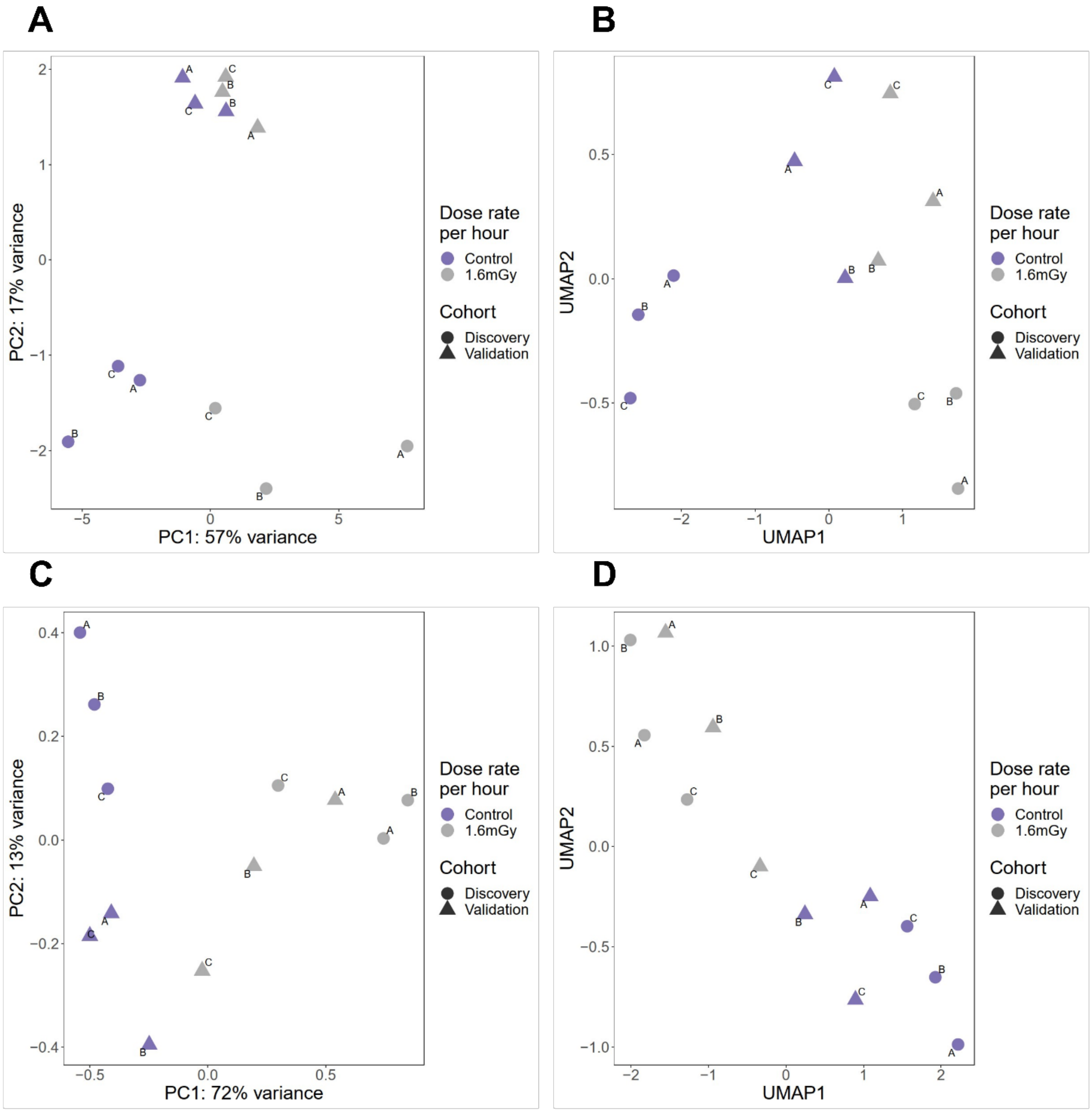

Supplementary Figure 1. PCA **(A)** and UMAP **(B)** analyses based on 389 selected genes with p-value < 0.05 (no correction for multiple testing) from a test comparing 1.6 mGy/h to control cells at 24 h in pooled data (discovery + validation cohorts) and analogous PCA **(C)** and UMAP **(D)** plots based on 8 genes with p-value < 0.001.

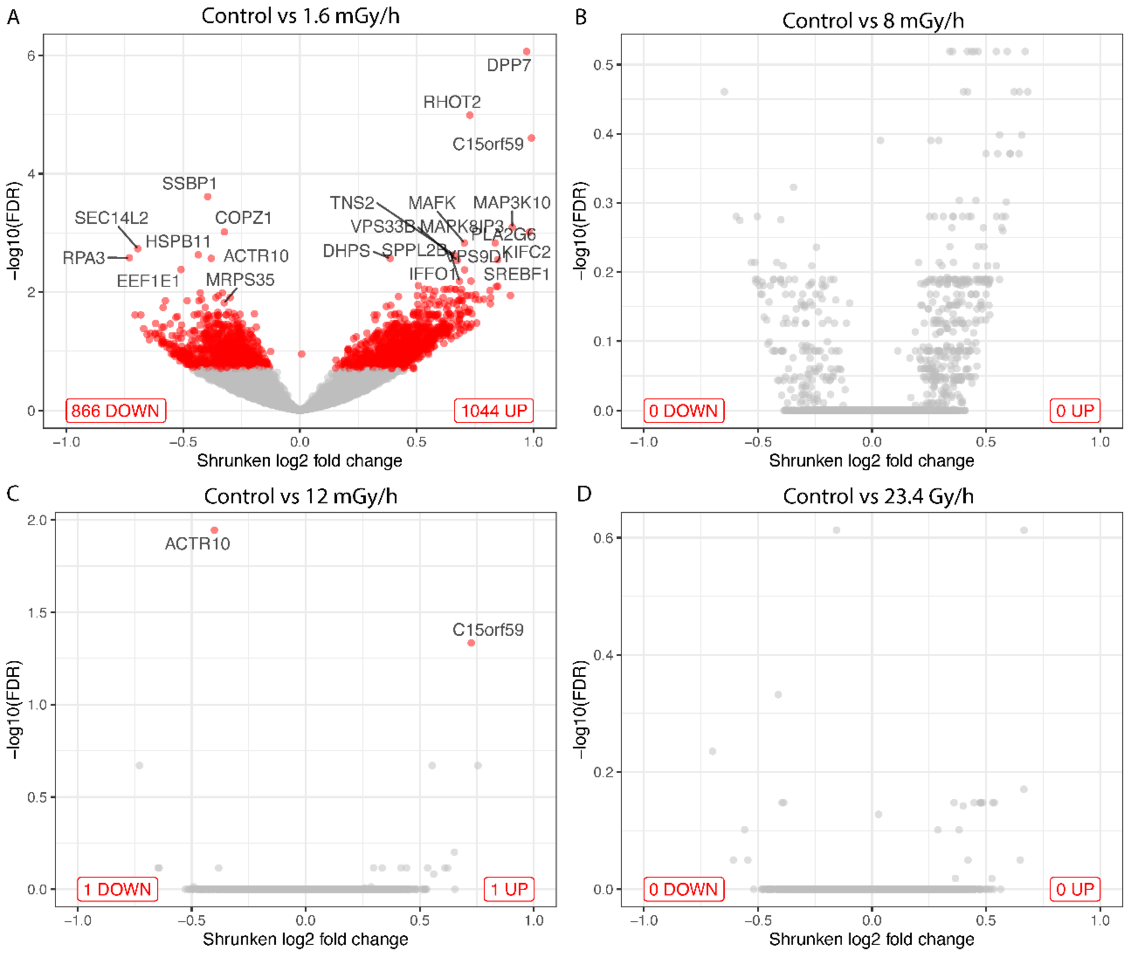

Supplementary Figure 2. Volcano plot representing the magnitude of gene expression changes and corresponding false discovery rate (FDR) for exposed samples as compared to control samples at the different dose rates (A-D: 1.6-12 mGy/h and 23.4 Gy/h, respectively) at 24 hours after exposure after removal of genes with low expression level from the discovery cohort.

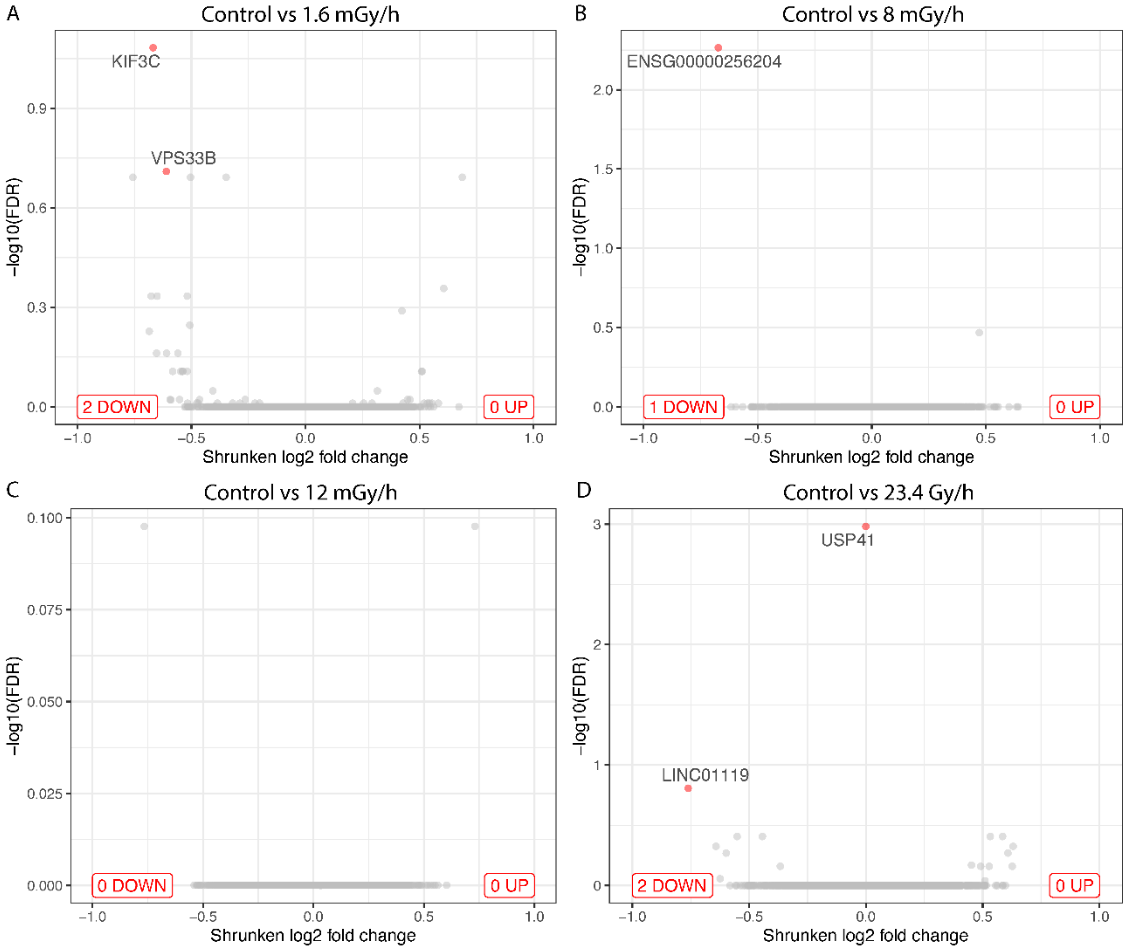

Supplementary Figure 3. Volcano plot representing the magnitude of gene expression changes and corresponding FDR on exposed samples as compared to control samples at the different dose rates (A-D: 1.6-12 mGy/h and 23.4 Gy/h, respectively) at 21 days after exposure after removal of genes with low expression level from the discovery cohort.

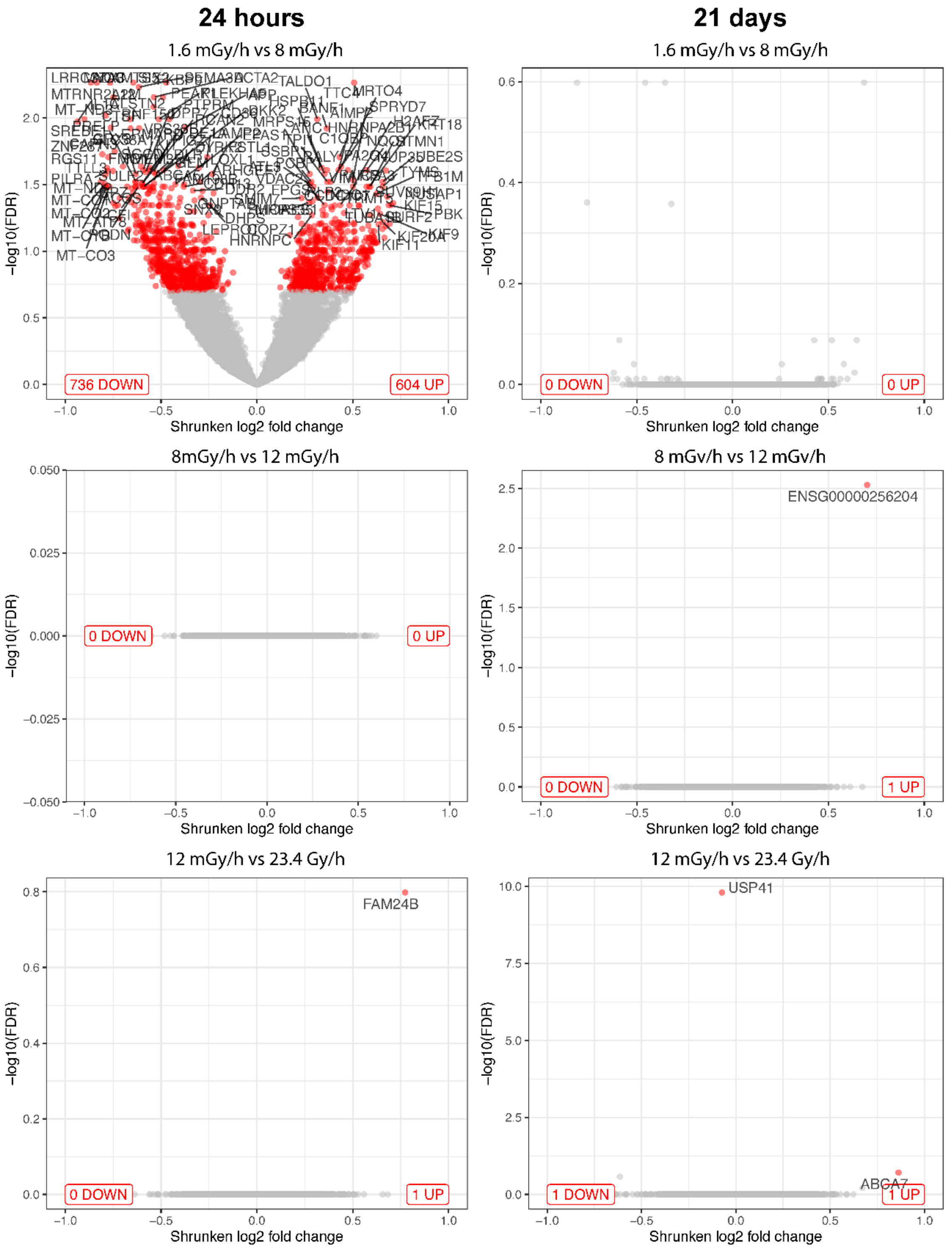

Supplementary Figure 4. Volcano plot representing the magnitude of gene expression changes and corresponding FDR on cells exposed to increasing dose rates as compared to the nearest lower dose rate, at 24 hours (left column) and 21 days (right column) after exposure after removal of genes with low expression level from the discovery cohort.

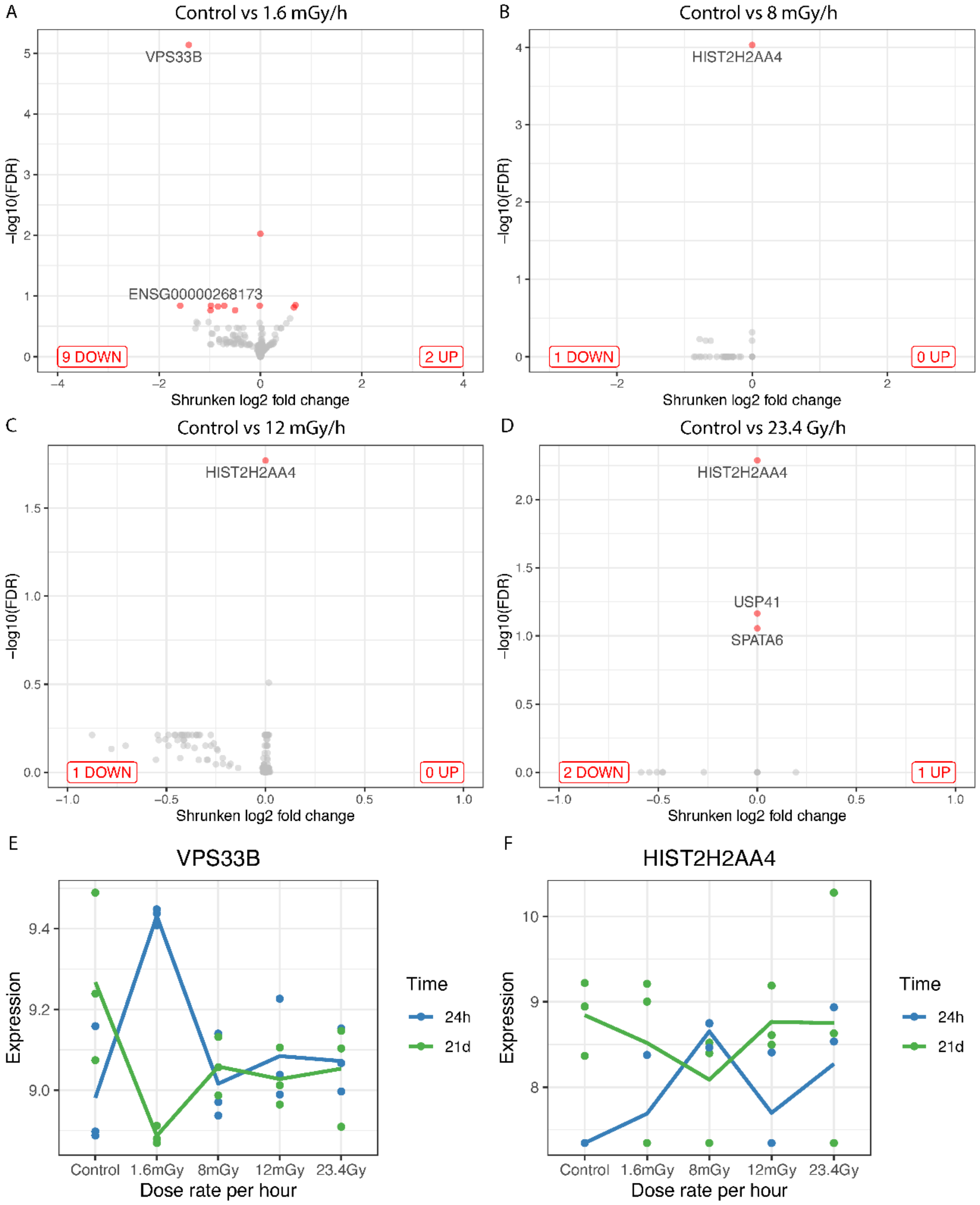

Supplementary Figure 5. Dose rate-Time interaction at the gene level at each dose rate as compared to control at 21 days as compared to 24 hours. A-D: Volcano plots for different dose rate-exposed cells as compared to control. E and F: Expression level of selected genes, i.e. VPS33B (E) and HIST2H2AA4 (F) for given dose rates and time points, i.e. 24 hours (blue) and 21 days (green) post-exposure.

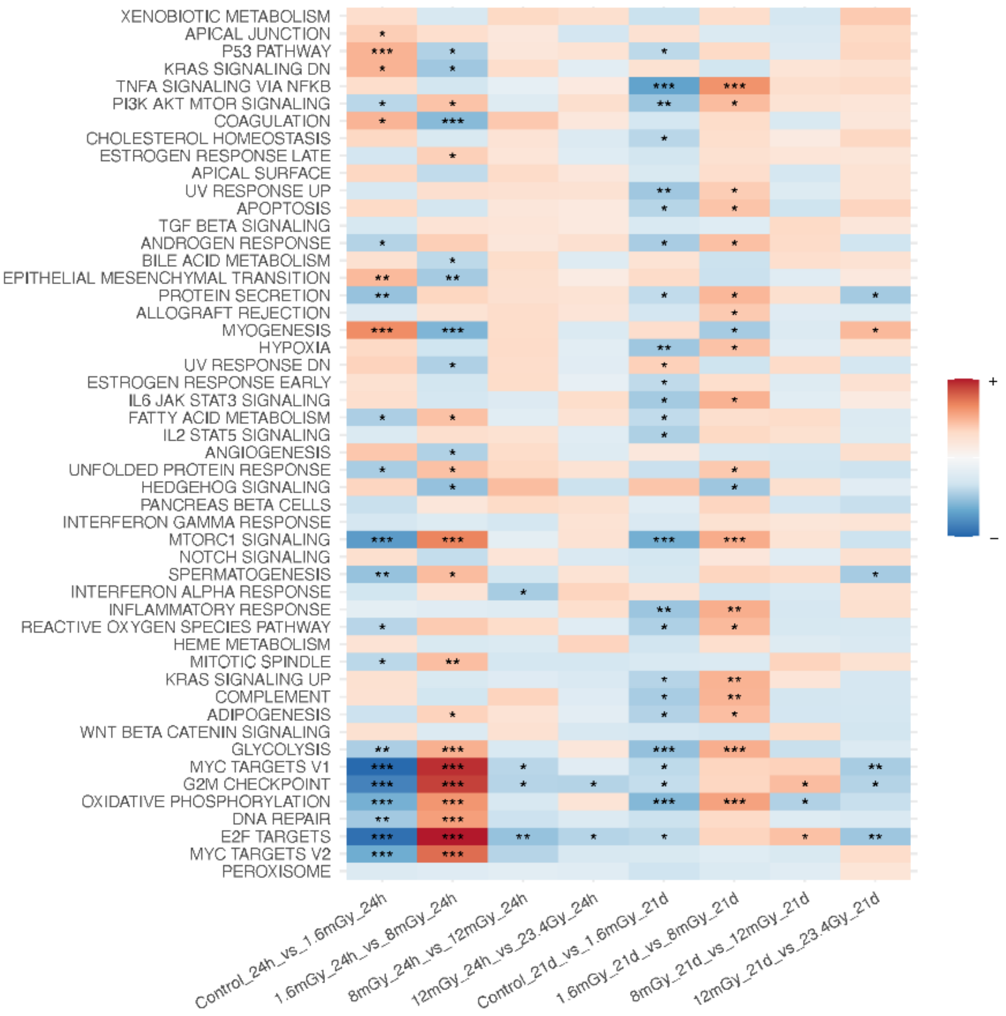

Supplementary Figure 6. Gene set enrichment analysis on MSigDB hallmark pathways after data filtration on each higher dose rate as compared to each lower dose rate for a given time point, i.e. 24 hours or 21 days. Red means upregulated at the higher dose rate as compared to the lower dose rate.

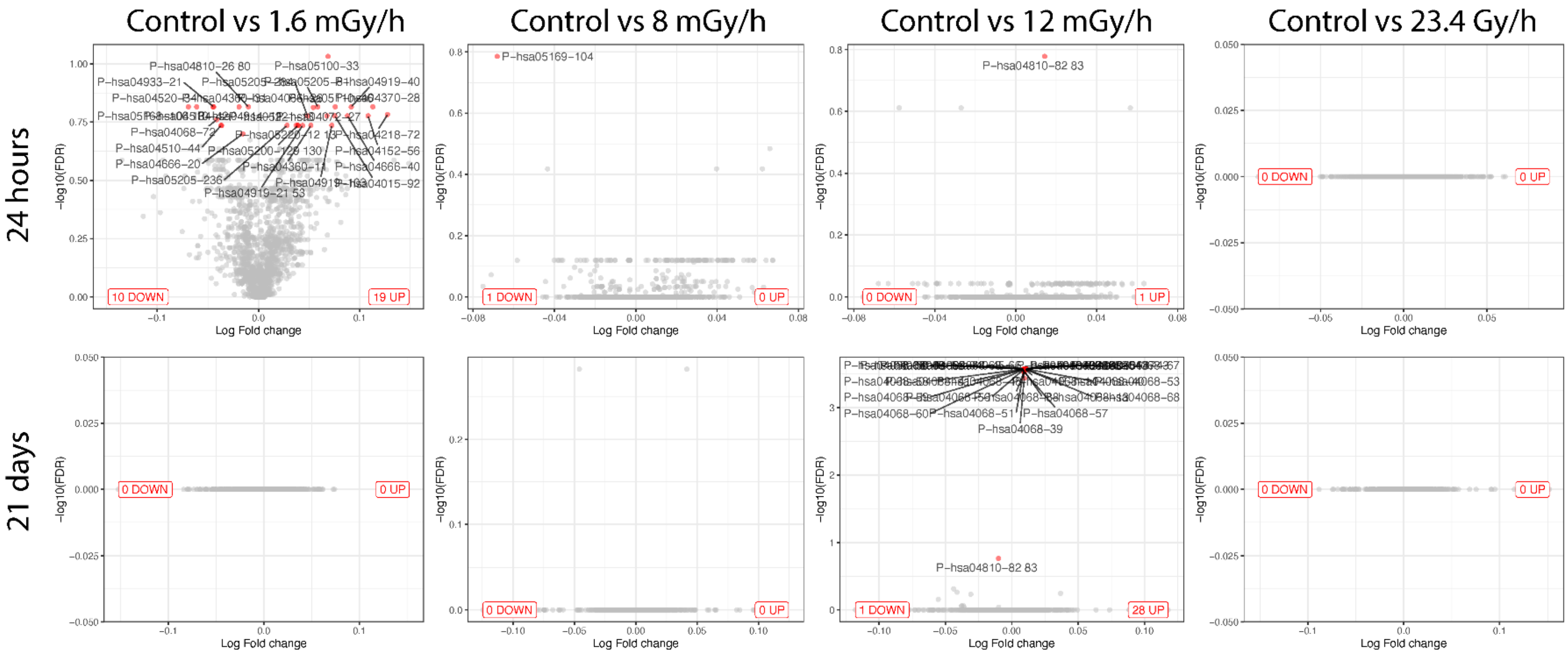

Supplementary Figure 7. Significantly different pathways based on Limma test results. For exposed cells at given dose rates as compared to control at 24 hours (top) and 21 days (bottom) after exposure.

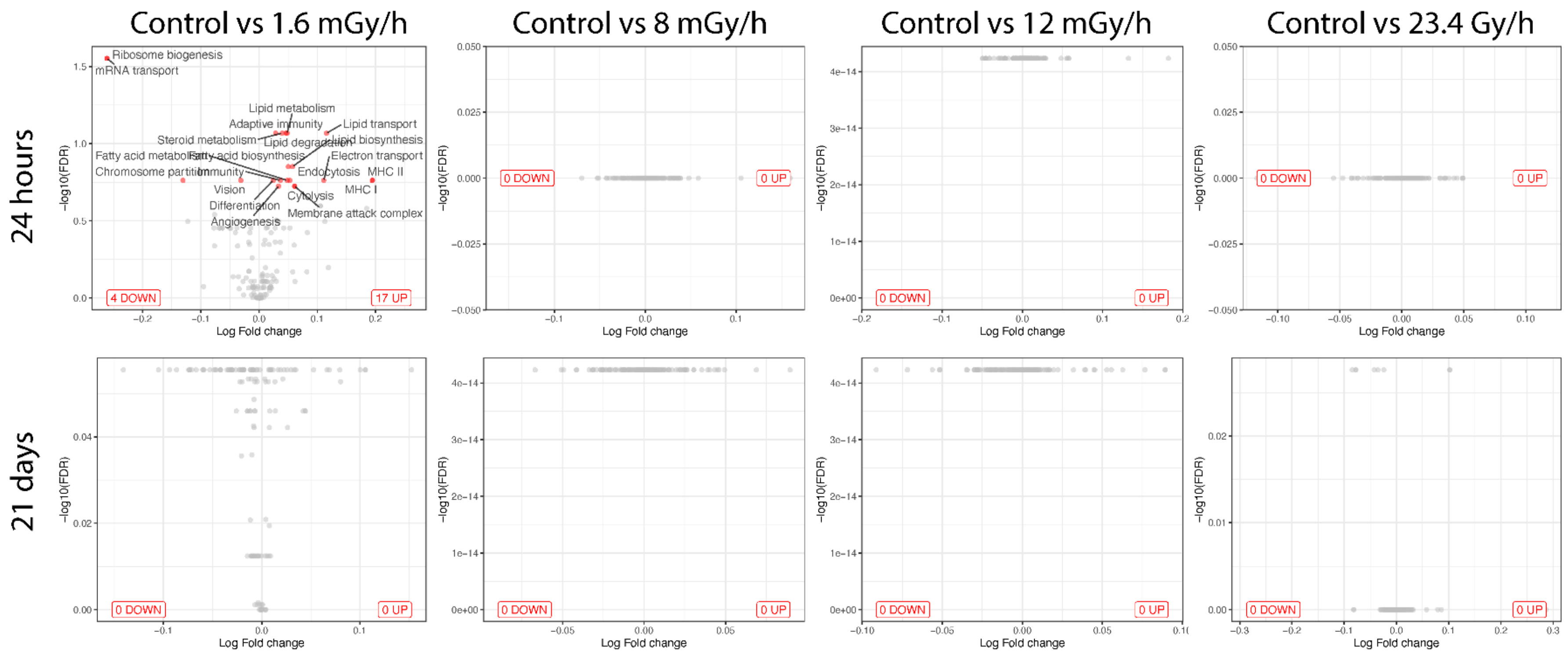

Supplementary Figure 8. Significantly different functions based on Limma test results. For exposed cells at given dose rates as compared to control at 24 hours (top) and 21 days (bottom) after exposure.

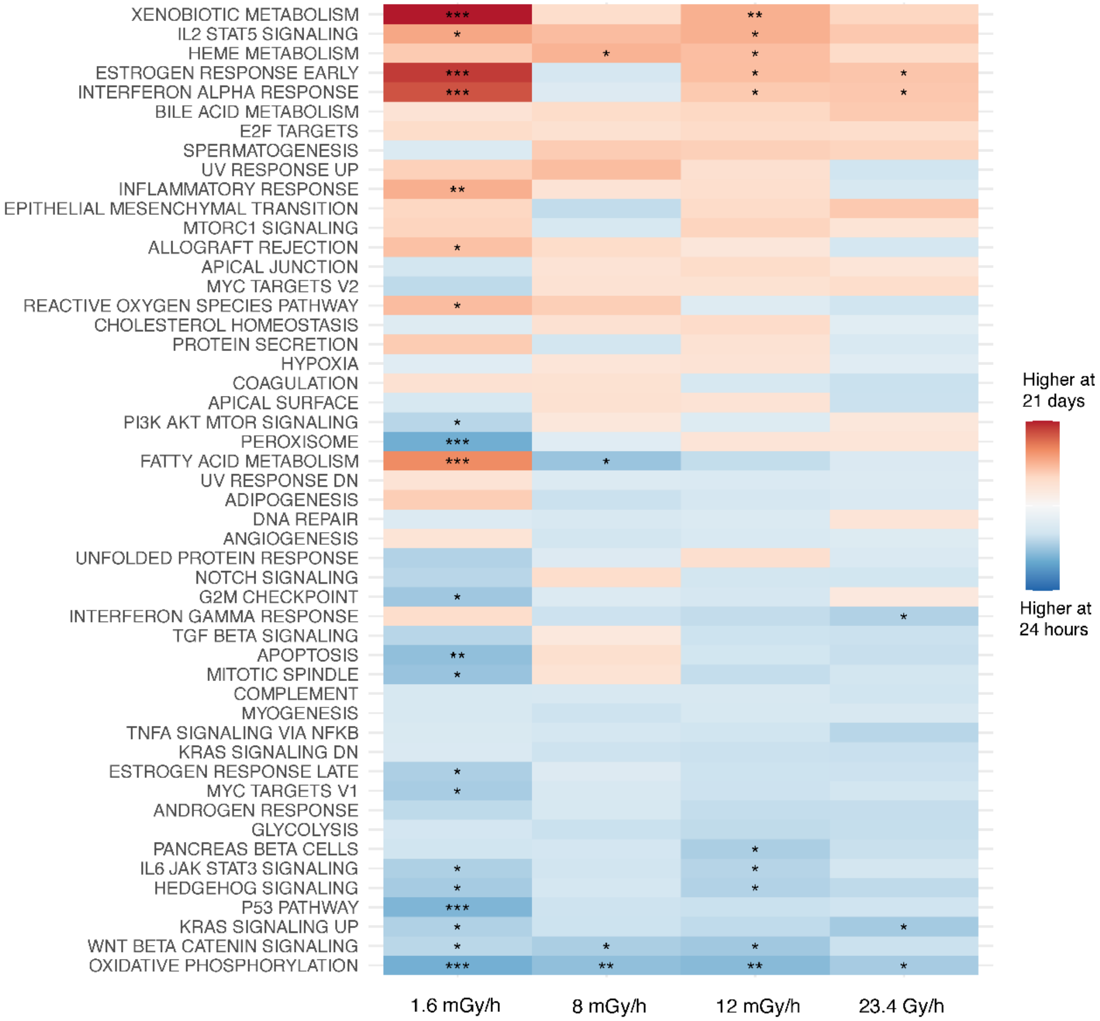

Supplementary Figure 9. Gene set enrichment analysis after data filtration of genes with differential expression between the two time points. Red means upregulated at 21 days as compared to 24 hours.

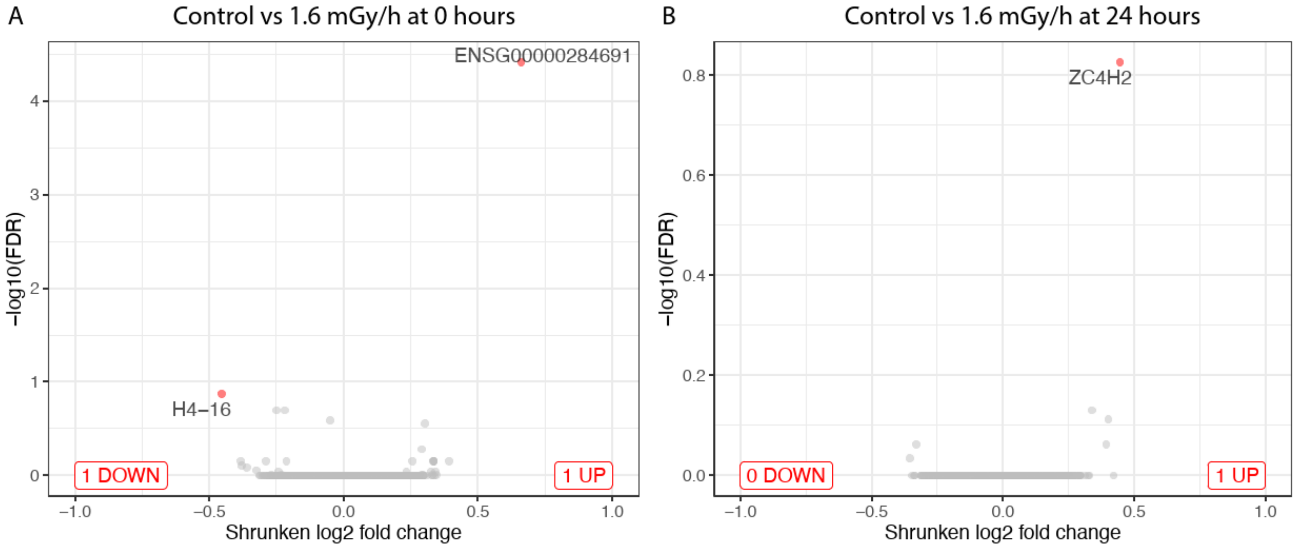

Supplementary Figure 10. A: Volcano plot for 1.6 mGy/h samples compared to controls at 0 hours in the validation cohort. B**:** Volcano plot for 1.6 mGy/h samples compared to controls at 24 hours in the validation cohort.

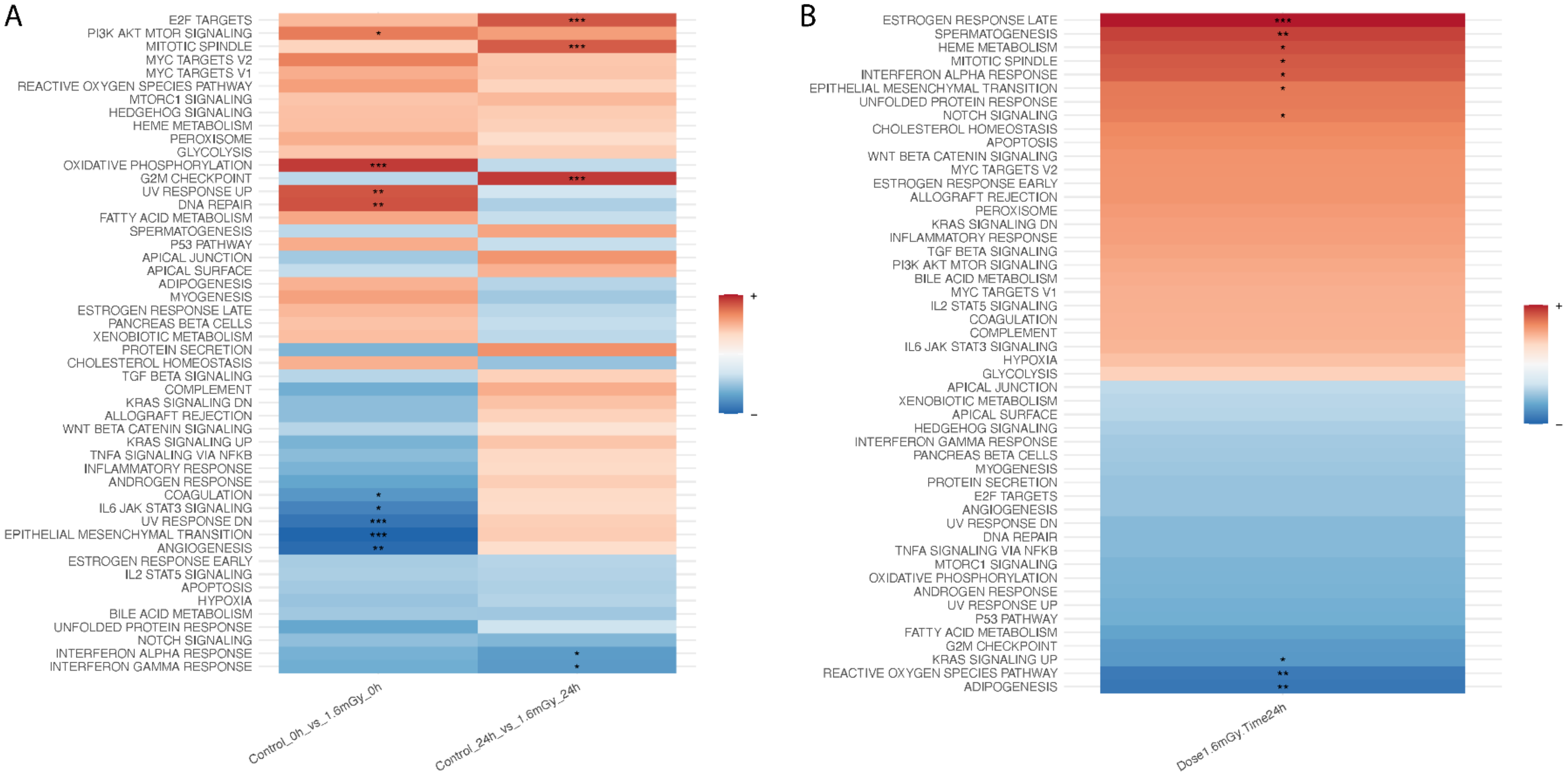

Supplementary Figure 11. Gene set enrichment analyses in irradiated sample as compared to control from the validation cohort for a given time point, whereby red means upregulated in exposed as compared to control (A) or for dose rate-time interactions, whereby 1.6 mGy/h relative to control (both at 24 h) is compared to 1.6 mGy/h relative to control (both at 0 h) (B). In B, red means upregulated at 24 hours.

A

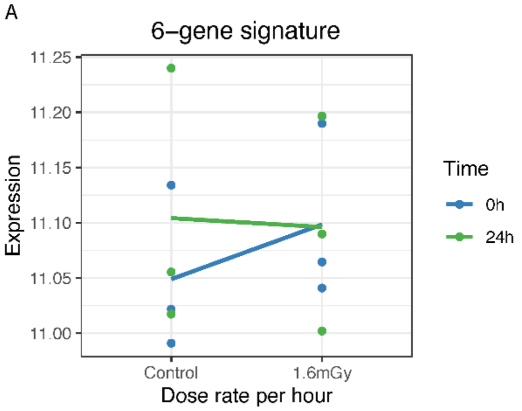

B

Comparison	Difference	Low CI	High CI	Adjusted p-value
1.6mGy:0h-Control:0h	0.049539	-0.19779	0.296871	0.915621
Control:24h-Control:0h	0.055274	-0.19206	0.302606	0.888179
1.6mGy:24h-Control:0h	0.047188	-0.20014	0.29452	0.925746
Control:24h-1.6mGy:0h	0.005735	-0.2416	0.253067	0.99984
1.6mGy:24h-1.6mGy:0h	-0.00235	-0.24968	0.244981	0.999989
1.6mGy:24h-Control:24h	-0.00809	-0.25542	0.239247	0.999553

Supplementary Figure 12. Dose Rate-Time interaction at the level of expression of the panel of genes in samples from validation cohort (A) with corresponding statistics (B).

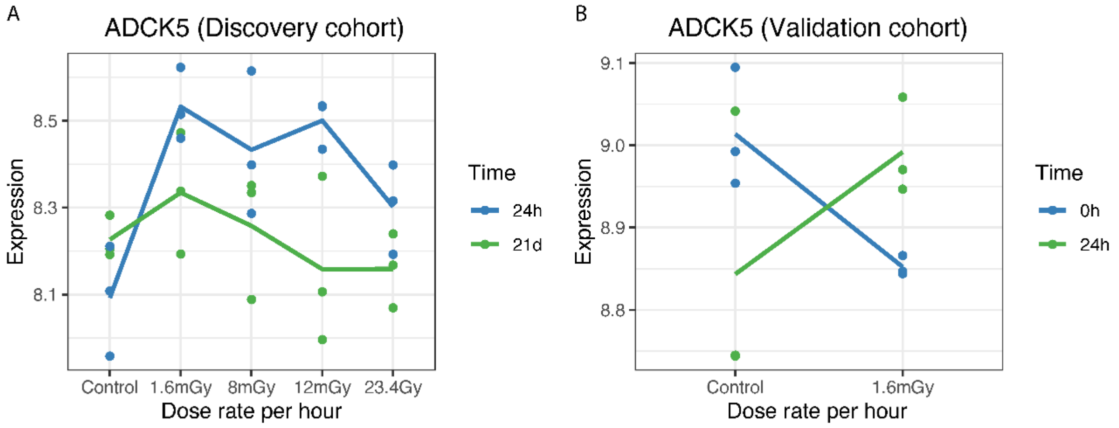

Supplementary Figure 13. *ADCK5* expression level at the different dose rates and time points in discovery (A) and validation (B) cohorts.

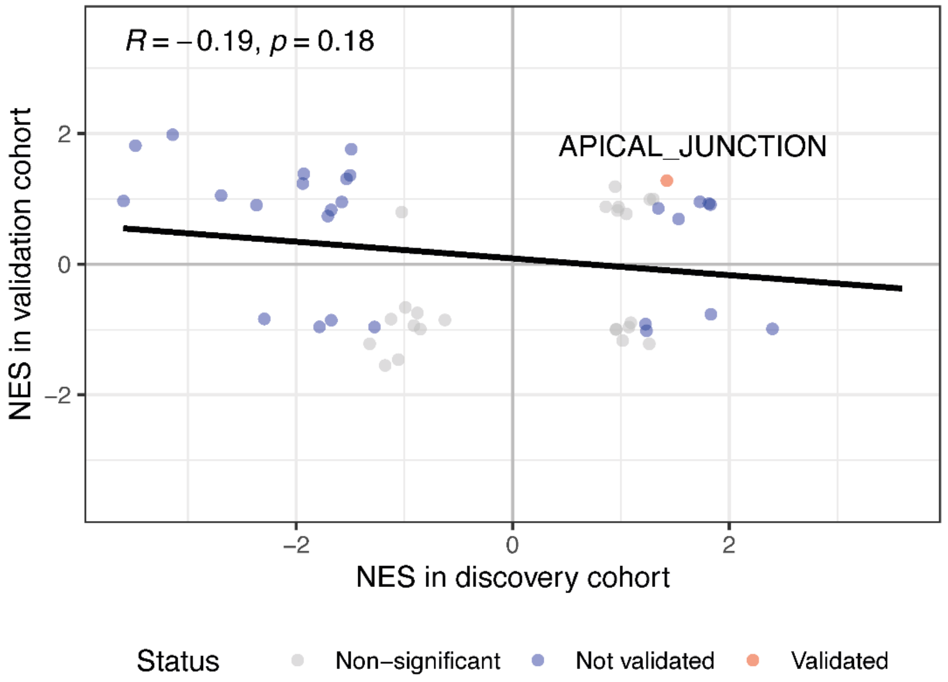

Supplementary Figure 14. Pathway level comparison of discovery and validation cohort for 1.6 mGy/h exposed cells as compared to control at 24 hours post exposure. Validation status of the different pathways (each circle represents one pathway) are indicated by color: non-significant (grey), not validated (blue) and validated (orange). NES: normalized enrichment score.

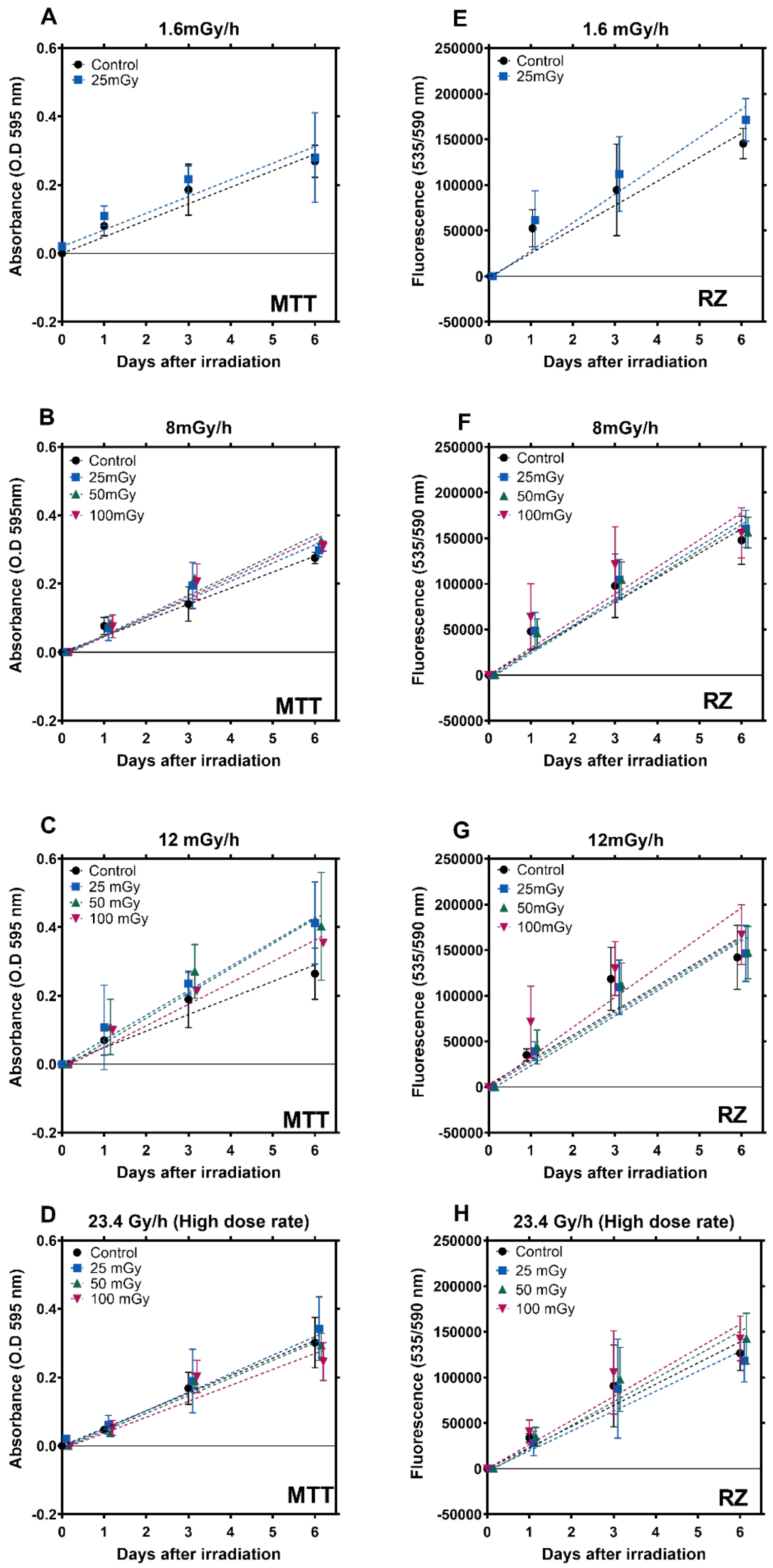

Supplementary Figure 15. Cell viability based on MTT (A-D) and resazurin (RZ, E-H) results from Figure 6 shown as a function of dose for each dose rate.

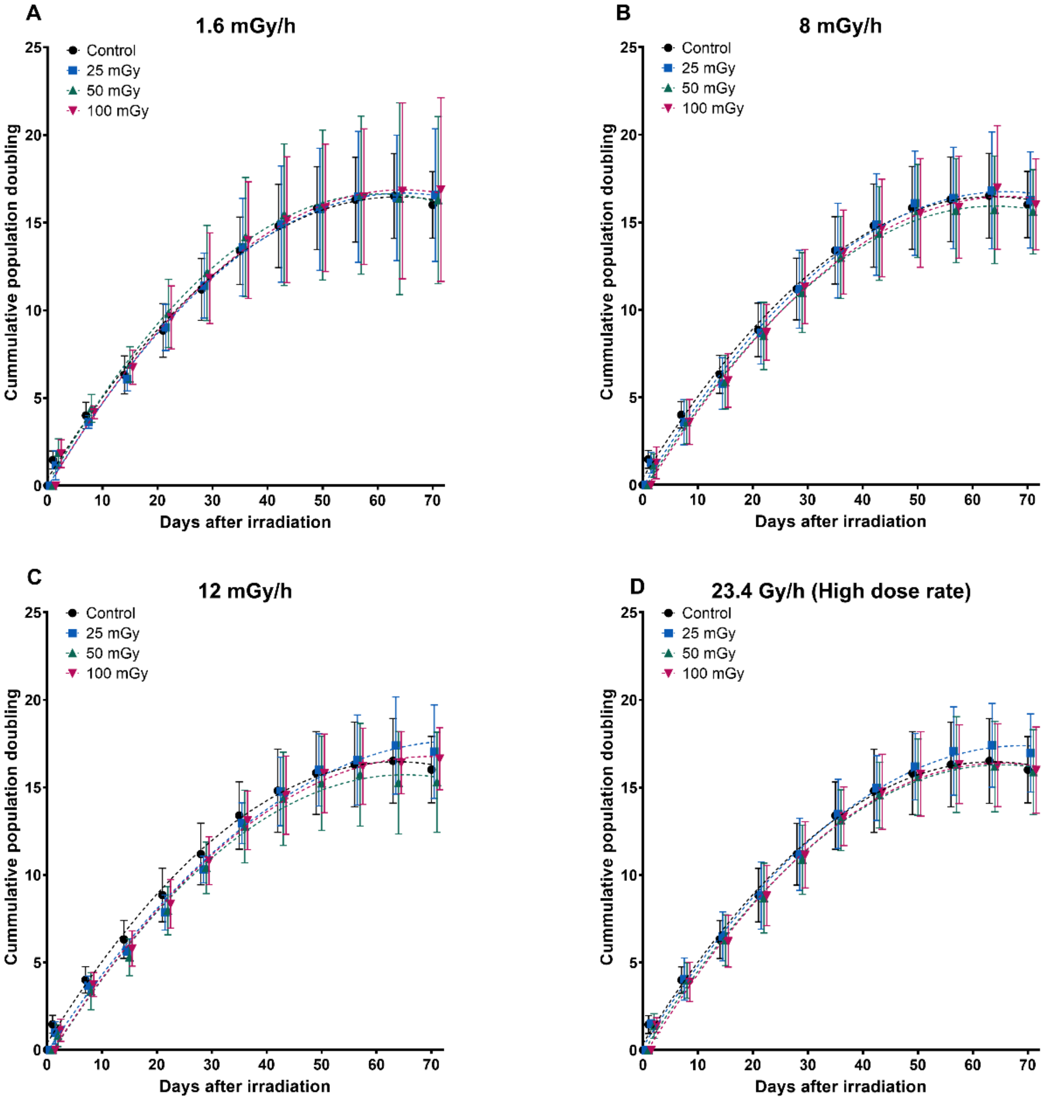

Supplementary Figure 16. Cell growth curves results from Figure 7 shown as a function of dose for each dose rate.
